# Life without Oxygen: Gene Regulatory Responses of the Crucian Carp (*Carassius carassius*) Heart Subjected to Chronic Anoxia

**DOI:** 10.1371/journal.pone.0109978

**Published:** 2014-11-05

**Authors:** Kåre-Olav Stensløkken, Stian Ellefsen, Olga Vasieva, Yongxiang Fang, Anthony P. Farrell, Lisa Olohan, Jarle Vaage, Göran E. Nilsson, Andrew R. Cossins

**Affiliations:** 1 Section for Physiology and Cell biology, Department of Biosciences, University of Oslo, Oslo, Norway; 2 Department of Physiology, Institute of Basic Medical Sciences, University of Oslo, Oslo, Norway; 3 Section for Sports Science, Department for Social Sciences, Lillehammer University College, Lillehammer, Norway; 4 Centre for Genomic Research, Institute of Integrative Biology, University of Liverpool, Liverpool, United Kingdom; 5 Department of Zoology, Faculty of Land and Food Systems, University of British Colombia, Vancouver, Canada; 6 Department of Emergency Medicine and Intensive Care, Institute of Clinical Medicine, Oslo University Hospital Ullevål, Oslo, Norway; Karlsruhe Institute of Technology, Germany

## Abstract

Crucian carp are unusual among vertebrates in surviving extended periods in the complete absence of molecular oxygen. During this time cardiac output is maintained though these mechanisms are not well understood. Using a high-density cDNA microarray, we have defined the genome-wide gene expression responses of cardiac tissue after exposing the fish at two temperatures (8 and 13°C) to one and seven days of anoxia, followed by seven days after restoration to normoxia. At 8°C, using a false discovery rate of 5%, neither anoxia nor re-oxygenation elicited appreciable changes in gene expression. By contrast, at 13°C, 777 unique genes responded strongly. Up-regulated genes included those involved in protein turnover, the pentose phosphate pathway and cell morphogenesis while down-regulated gene categories included RNA splicing and transcription. Most genes were affected between one and seven days of anoxia, indicating gene regulation over the medium term but with few early response genes. Re-oxygenation for 7 days was sufficient to completely reverse these responses. Glycolysis displayed more complex responses with anoxia up-regulated transcripts for the key regulatory enzymes, hexokinase and phosphofructokinase, but with down-regulation of most of the non-regulatory genes. This complex pattern of responses in genomic transcription patterns indicates divergent cardiac responses to anoxia, with the transcriptionally driven reprogramming of cardiac function seen at 13°C being largely completed at 8°C.

## Introduction

Vertebrates in general are highly dependent on aerobic metabolism and most suffer profound negative effects of environmental or tissue hypoxia. The freshwater crucian carp (*Carassius carassius*) is unusual in tolerating the complete absence of oxygen for several months at low temperatures [1]. To maintain energy balance in the absence of oxidative phosphorylation, the fish is thought to up-regulate glycolysis [2], while overall metabolic rate is moderately depressed [3]. Thus, brain activity is reduced in anoxia, as indicated by reduced heat production [4] and suppression of sensory modalities like vision [5] such that ATP demand could be supplied entirely by glycolysis. Lactate produced by the brain and other tissues is converted to ethanol in skeletal muscle, avoiding the severe acidosis associated with anaerobic glycolysis [6].

Curiously, in contrast to mammalian hearts, cardiac activity in carp is fully maintained during prolonged anoxia [7] to sustain blood transport of glucose to fuel glycolysis in all tissues, of lactate to skeletal muscle, and of ethanol to the gills [8]. The lack of detrimental effects of anoxia exposure on the crucian carp heart contrasts with mammalian hearts, which show extensive and irreversible injury including myocyte cell death following interruption of blood flow (ischemia) and after the subsequent reperfusion [9]. This makes the anoxic crucian carp heart a particularly interesting model to understand how evolution has solved the problem of anoxic survival.

Ischaemia tolerance in the mammalian heart is improved by short ischemic ‘preconditioning’ episodes [10] and the molecular correlates of this protective response are central to contemporary cardiac research [11]. Protective pathways converge on the mitochondria [12], [13], which in turn controls myocyte cell death [14], [15], and regulation of the mitochondrial permeability transition pore (MPTP) may underpin responses to ischemia and preconditioning [9], [13], [16]. Also enhanced glycolysis under hypoxia might underpin the enhanced ischemia tolerance of neonatal mammalian hearts [17]. Ischemia tolerance in adult rat hearts increased after chronic exposure to hypoxia [18], while the hearts of zebrafish (*Danio rerio*) held under chronic hypoxia (10% of air saturation for 21 days) displayed a shift towards anaerobic metabolism in gene expression profile [19]. These hypoxic events have comparatively little effect upon the molecular responses of the crucian carp, such as in AMP-activated protein kinase (AMPK) phosphorylation, indicating no shift in the AMP/ATP ratio [20]. Given the extremely high oxygen affinity of crucian carp haemoglobin, this is perhaps not surprising and so the molecular responses to anoxia are all the more important.

However, anoxia survival in the crucian carp has been linked to responses induced in the brain by the inhibitory neurotransmitter, gamma-amino-butyric acid (GABA) [21], possibly linked to inhibition of GABA re-uptake [22], and in the excitatory N-methyl-D-aspartic acid (NMDA) receptor subtype composition, possibly resulting in decreased excitability [23]. The protein kinases AKT and AMPK, both linked to glucose metabolism, were regulated at a post-translational level in both brain and heart [20], and blocking AMPK with compound C increased ethanol production, consistent with a role for AMPK in whole body metabolism [20].

The true anoxic survivors, such as crucian carp and some freshwater turtles, can withstand chronic anoxia at cold temperatures [24] suggesting that winter cooling may act as a seasonal cue for developing the anoxia-tolerant winter phenotype [25]–[27]. In the common carp (*Cyprinus carpio*), chronic cooling transformed the transcriptome of all tissues examined [28] and the same may be true for the crucian carp [29], [30]. We hypothesized that the overall transcriptional effect of anoxia would be smaller at 8°C than at 13°C and that a reduction in temperature alone might result in preconditioning of the crucian carp heart for exposure to anoxia. Here we describe a transcriptomic approach to testing these ideas, based on new statistical techniques and increased biological replication, to the screening of differentially expressed genes at low fold-change responses.

## Material and Methods

### Experimental animals

Crucian carp were caught in Tjernsrud pond (59°55°17.563 N, 10°36°32.773 E), a public pound with no specific permit required, Oslo community, Norway, in June. Crucian carp is not an endangered species in Norway. They were transferred to and maintained in 750 litre tanks continuously supplied with de-chlorinated, aerated Oslo tap water at ambient temperatures, with a 12 h/12 h photoperiod and *ad libitum* feeding with a commercial carp food. Water temperature varied seasonally; being 13 °C during the first experimental series in August, and 8 °C during the second series in November. Fish were held at these temperatures for at least two weeks before the experiments were conducted. All experiments were approved and conducted in accordance with the Norwegian Animal Health Authority.

### Experimental design

Crucian carp (32 individuals, weighing 40±16 g) were randomly selected from the holding tank and placed in a circular 25 litre PVC tank with a flow-through system (0.5–1.0 ml/s) sealed with an air-tight lid. Fish were conditioned to the tank for 24 hours prior to anoxic exposure. Anoxia (<0.01 mg O_2_/L) was obtained by bubbling the incoming water with nitrogen in a 1.5 m Plexiglas column (or with air during reoxygenation). Oxygen concentrations and temperatures were continuously monitored with a galvanometric oxygen electrode, Oxi 323 (WTW, Weilheim, Germany). Normoxic controls were obtained by putting an equal number of fish in an identical tank continuously bubbled with air. After exposure, fish were stunned with a sharp blow to the head, and killed by bisection of the spinal cord and dorsal aorta, followed by cardiac ventricular tissue sampling. Two similar exposure series were conducted: one at 13 °C and one at 8 °C. Each series contained the following four groups (n = 8 in all groups):

1. Normoxic controls at 8°C (8N7) and at 13°C (13N7).
2. One day anoxia at 8°C (8A1) and at 13°C (13A1).
3. Seven days anoxia at 8°C (8A7) and at 13°C (13A7).
4. Seven days anoxia followed by seven days of re-oxygenation at 8°C (8R7) and at 13°C (13R7).

### RNA extraction and cDNA synthesis

Total RNA for microarray and real-time RT PCR experiments was extracted from crucian carp ventricles using 15 µl TRIzol per mg tissue. The extractions were performed as described previously [31]. Tissue homogenates for further analysis were limited by the smallest available tissue sample in each of the two experiments (5 mg and 17 mg for 8°C and 13°C, respectively). The quality and quantity of the total RNA was assessed using a 2100 Bioanalyzer (Agilent Technologies, Palo Alto, CA, USA) and a NanoDrop ND-1000 UV-Vis Spectrophotometer (NanoDrop Technologies, Rockland, DE, USA). For the microarray and real-time RT PCR experiment, respectively, 5 and 1 µg total RNA was reverse transcribed using oligo(dT)_18_ and Superscript III (Invitrogen).

### Microarray analysis

**Figure 1:**
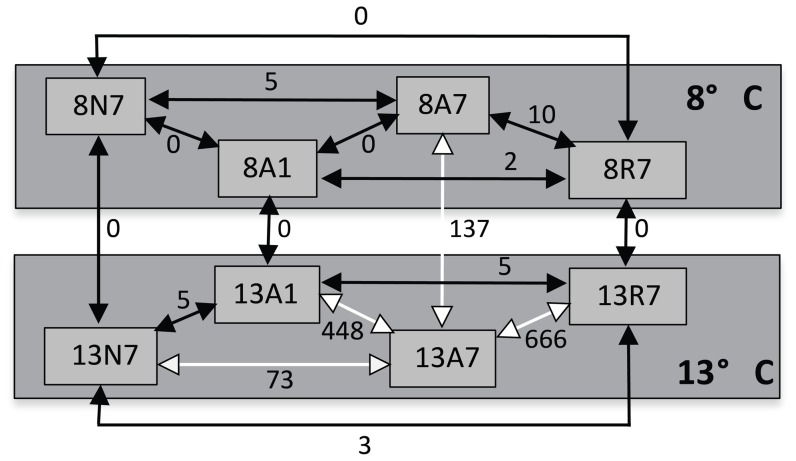
The hybridisation design employed in this experiment. Each double-headed arrow represents two 2-dye-swapped microarrays contrasting the indicated treatments. Each RNA sample was taken from a different carp specimen and thus represented a biologically independent sample. A total of 32 arrays and 64 RNA samples were used (12 arrays were used to compare specimens at 8°C, a further 12 compared those at 13°C and 8 arrays compared 8 with 13°C). Thus, each condition was sampled with 8 independent specimens. The numbers adjacent to the arrows indicate the number of differentially expressed genes detected for the indicated contrast (FDR <5%). The white arrows indicate the contrasts possessing the majority of differentially expressed genes, all of which were connected to the A7 treatment (anoxia for 7 days).

The common carp microarray (version 5) used in these experiments has been described recently [32]. It contains 19,584 PCR-amplified cDNA clones printed on to Corning GAP II glass slide using a BioRobotics TAS printing robot. We used a dye-balanced design for 2-colour array hybridisations with 8-fold biological replication, as illustrated in Fig 1. cDNA from two compared samples were separately labelled with either cyanine-3 and cyanine-5 dyes (GE Healthcare, Amersham), combined and applied under Lifterslips (Thermo Scientific) for an overnight hybridisation. Images were collected using a Genepix laser scanner and intensity values extracted using Genepix 3 software. Gene expression profiles were calculated within each treatment group at (i) 8°C, (ii) 13°C, and (iii) for comparisons between each treatment group at 8°C and 13°C. This gave a total of 64 RNA preparations comprising eight treatments each with 8-fold biological replication. Raw data was deposited in the ArrayExpress repository (accession E-MTAB-2516).

### Statistical analysis of microarray data and interpretation of differentially expressed cDNA clones/genes

The measured data was normalised through variance stabilised normalisation [33], followed by a loess-based spot intensity dependent dye-bias correction [34] and a spatial bias correction. The normalised data was then formulated by a linear model involving canonical parameters [35]. The Maximum Likelihood Estimation (MLE) of the parameters was generated by fitting the model to data. The significance of differentially expressed (DE) cDNA clones on the array was determined using the t-test. The cDNA clones were BLAST annotated as previously described (55). In cases where different clones gave the same BLAST annotation, they were averaged even though the existence of different paralogs was not ruled out (see *Methodological considerations*).

The multiple testing problem was handled by using q-values [36] and DE genes were extracted by controlling the FDR (False Discovery Rate; [37], [38]) at 5%, i.e. q<0.05. The identified DE genes were clustered across all contrasts by the K-means method and the gene expression properties of all DE genes was visualised using heat-maps.

### Interpretation of gene lists

We explored the significance of categories of the Gene Ontology (GO) ‘Biological Process’ domain using two methods. The first was a statistical test of over- or under-representation of GO categories in DE gene set by a hyper geometric test [39], [40]. The significant GO terms were extracted using a *q*-value cut-off at 5%.

Second, we developed a test based the on the rank order of *p*-values for genes within specific GO categories. Because the results for the first method were dependent to some extent upon the number of DE genes, we sought a separate test of the significance of biological process categories. For this, a list comprising all microarray gene probes with a GO annotation was ranked by *p*-value, giving the rank set R. The genes associated with any GO category were selected and the rank order was taken to form the query gene set. The rank value can be viewed as a set of random samples from R when the null hypothesis is true. We calculated the mean rank for each query gene set and used this as test statistic. When the size of a query gene set was one or two, we conducted the test based on a distribution calculated exactly from a discrete uniform distribution. When the size of the query gene set>2, we transformed the mean rank into a statistic which approximately follows the standard normal distribution. Let N be the number of genes measured on the microarrays, n_i_ be number genes of query gene set i and r_i_ be the mean ranks of query gene set i, the transformation is: (r_i_-c)/s_i_, where c = (N+1)/2 and s_i_ is the square root of (N- n_i iii_)(N+1)/12/n_i ii_. Finally, the *q*-values were calculated from p-values to handle the issue of multiple testing. Significant GO terms were identified using a cut off of q = 0.1.

This *p*-ranking method has several advantages over other popular methods (i.e. [39]). These are that (i) it is simple both computationally and in application, (ii) the results are unique and not affected by the means of extracting DE genes, and, (iii) it can include the terms associated with weakly responding genes which are typically not included in DE gene lists.

### Pathway analysis in the anoxic crucian carp

The DE gene list was interpreted using Ingenuity Pathway Analysis (IPA; Ingenuity Systems, Redwood City, CA) software. IPA integrates gene expression data within the context of known biological pathways and processes that provides a better understanding of the biological impact of responses in relation to higher-order physiological and disease processes. The list of human orthologs of the DE carp genes derived *via* bi-directional blast of the transcripts against human genome was used as an input to IPA's database “Core Analysis”. Gene Symbols have been used as Identifiers and the Ingenuity Knowledge Base gene set as a reference for a pathway analysis. ‘Canonical Pathways’ and ‘Networks’ were ranked by IPA according to potential significance of their coverage. The significance of the association between the data-set and the canonical pathway was measured in two ways: (a) By calculating a ratio of the number of molecules from the data set that map to the pathway divided by the total number of molecules that map to the canonical pathway, and (b) By Fisher's exact test applied to calculate the probability (p value) that the association between the genes in the data set and the canonical pathway is explained by chance alone.

### Methodological considerations

Here we used a custom-made microarray constructed using cDNA clones of the common carp to probe mRNA targets from crucian carp. Although this is a better option than commercially available zebrafish arrays, poorer hybridization due to sequence mismatches between probe and target is possible when dealing with two different species. However, comparative studies have shown that excellent hybridisation signals can be obtained using heterologous arrays [41] even between distantly related fish species [42]. Of course, the close phylogenetic relationship of common carp and crucian carp favours this approach. Furthermore, both crucian and common carp have experienced recent genome duplication [43], and many duplicated genes are retained albeit with divergence in tissue specificities and perhaps functions [44], [45]. In other cases, the differential loss of one or other duplicate between the two carp species might occur though current data is not sufficiently complete to identify which has occurred, thus orthology cannot be assured. We have identified a number of cases where multiple probes for a given BLAST identity displayed divergent expression responses; these may well represent paralogs.

## Results

### Gene expression profiles following anoxia treatment

**Figure 2:**
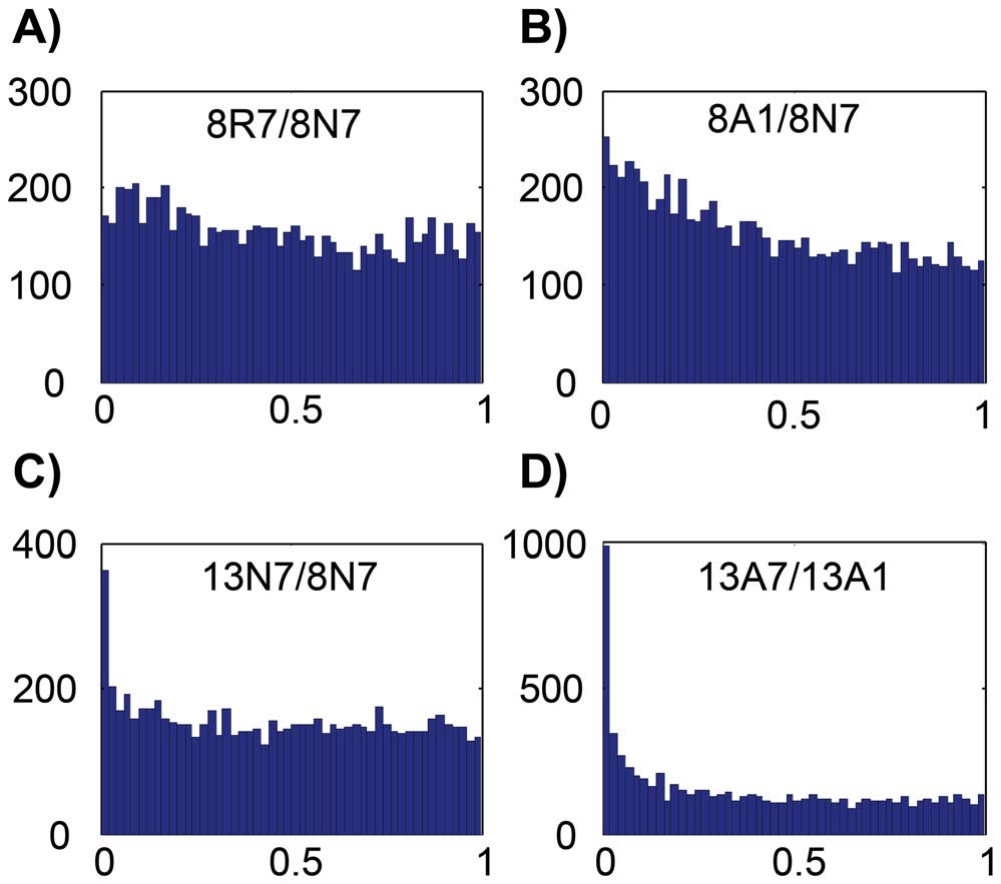
Bar-charts displaying types of *p*-value distributions for contrasts between selected treatments. A random distribution would be represented by an equal representation of *p*-values across the full range, generating a flat histogram, as in the 8R7/8N7 contrast, top left panel. In some contrasts, the proportion of genes with p<0.05 was much greater indicating a non-random distribution, as in the 13A7/13A1 contrast, bottom right panel. The proportion of these gene probes that were randomly included in this category (i.e. the false discovery rate) was estimated by backwards extrapolation of the horizontal line above *p*>0.2. The two other contrasts represent intermediate conditions in which DE genes were evident but were difficult to extract as significant due to large proportions of false positives.

We contrasted the treatment groups using the design shown in Figure 1 based on a series of comparisons using two-colour arrays. The statistical model provided a log_2_ fold-change value for each gene probe between each of the indicated contrasts, along with the associated *p*- and *q*-values in order to determine the statistical significance of the difference. Figure 2 illustrates the distribution of *p*-values for all of the 20 K gene probes represented on the microarray for selected contrasts. A flat profile (e.g. Figure 2A) indicates a random distribution of *p*-values, such that all genes with *p*<0.05 were likely to be false positives. For other comparisons, we noted an increase in the number of gene probes with *p*<0.05 (e.g. Figure 2B–D). The proportion of false positives in this *p*<0.05 group was estimated by back-extrapolation of the horizontal line covering *p*-values across the full range. Figure 2B displays the 8A1/8N7 contrast with intermediate levels of true positives; although there was an increase in the proportion of gene probes at low *p*-values, confidence in assigning differential expression was low. In the temperature contrast (13N7/8N7, Figure 2C), there was a clear over-representation of genes with p<0.05, but most of these were rejected by statistical tests. In contrast, the 13A7/13A1 comparison (Figure 2D) showed a large number of gene probes with low *p*-values, well above that expected by chance alone. We have also assessed the importance of gender in the effects of anoxia/reoxygenation treatment (data not shown) and found no measureable effect either on its own or in combination with anoxia treatment.

A false discovery rate criterion of *q*<0.05 resulted in a list of 2,070 differentially expressed (DE) gene probes with significance in at least one contrast. These comprised 844 unique genes distributed across contrasts indicated in Figure 1. At 8°C, the transition of crucian carp from normoxia to anoxia (i.e. 8A1, 8A7) followed by reoxygenation (8R7) was linked to very few or no DE genes. By contrast, at 13°C we found 777 DE genes when comparing the A7 group with the N7, A1 or the R7 group. Comparisons of the 8°C with the corresponding 13°C groups revealed differences only for the 7-day anoxia treatment, with 67 unique genes significantly regulated.

**Figure 3:**
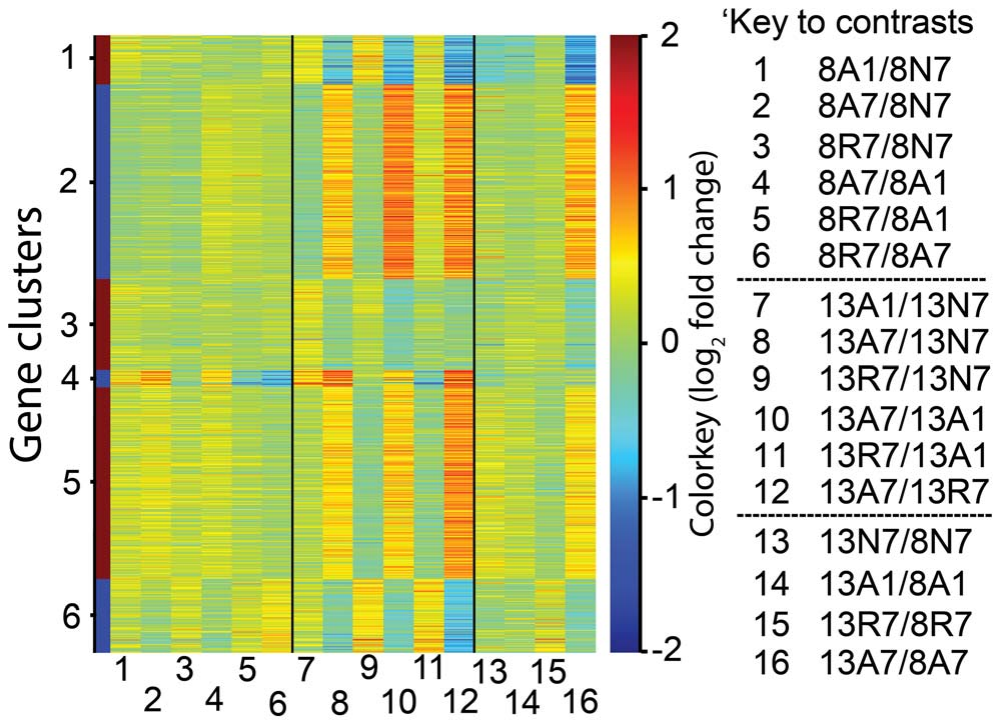
Heat map representing the log_2_ fold-change values between contrasted treatments for the 844 genes found to be differentially expressed in at least one of the contrasts. Each of the 16 columns represents a different contrast between treatment groups, as indicated in the key to the right, and as illustrated in Figure 1; note that the 13A7 group was the denominator in most of these contrasts. The value of fold-change between contrasted treatments for each gene was indicated by the coloured scale, with red indicating a value>1 and blue indicating a value <1. Genes were clustered into 1-6 groups using the K-means technique, each possessing broadly the same profile across all contrasts.

Figure 3 illustrates the overall response profile as a colour-coded ‘heat map’ across 16 different contrasts, as indicated in Figure 1 for all 844 DE genes. These were grouped using the K-means technique into six clusters (C1–C6) comprising 68, 265, 124, 24, 262 and 101 genes, respectively. Comparisons of the treatment groups at 8°C (comparisons 1-6) displayed low fold-change differences, as indicated by the turquoise or pale yellow colours. However, contrasts 8, 10, and 12, showed much richer yellow-red and blue colours, indicating more substantial fold-change values. Three gene clusters (i.e., C2, C4 and C5) with red colours included genes with up-regulated expression in the A7 group compared to the other three treatment groups, and three gene clusters (i.e., C1, C3 and C6) with blue colours, indicating down-regulated gene expression. The contrasts between the two temperatures indicated little difference in gene expression, except that for 8A7-13A7 were 67 unique genes were significantly changed.

**Figure 4:**
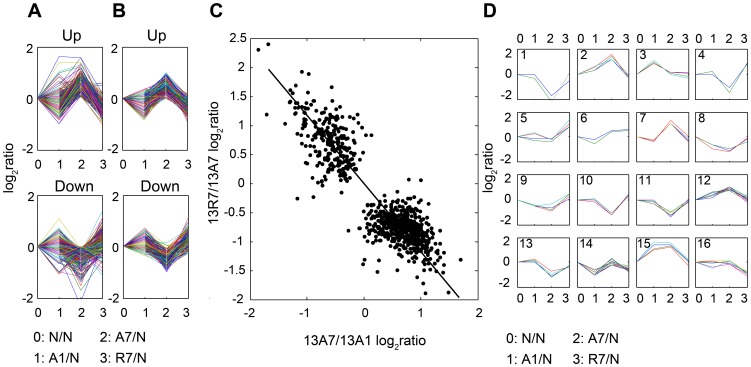
Comparison of expression profiles for contrasts between treatment groups at 13°C. Panel A indicates those genes up-regulated (upper) and down-regulated (lower) by anoxia treatment. Panel B presents the same profiles following removal of the outlier genes as described in the Methods section. Panel C shows that the anoxic response (A7/A1 contrast) was inversely correlated with the subsequent reoxygenation response (R7/A7 contrast). Panel D illustrates the diverse profiles for 75 outlier genes grouped into 16 clusters.

We then explored the time-course of the gene responses and the quantitative relationship between the effects of anoxia, the subsequent re-oxygenation treatment, and the relationship between the two, as illustrated in Figure 4 for 777 responding genes at 13°C. The principal transcriptional response occurred from one to seven days of exposure to anoxia. Most of the anoxia-induced changes were restored upon re-oxygenation (Figure 4A–C). Panel A depicts separately the responses of the grouped up- and down-regulated DE gene clusters shown in Fig 3 at 13A1, 13A7 and 13R7, all relative to 13N7 controls. From these genes we extracted 75 outlier genes using a mathematical separation technique (Figure 4D), leaving two large groups with 13A7 up-regulated (478 genes, panel B, upper) and down-regulated (224 genes) profiles (panel B, lower). In both cases, the 13R7 treatment restored gene expression to levels close to that at 13A1. This point is made quantitatively in Figure 4C, where the responses to seven days of anoxia (i.e. 13A7/13A1) were negatively and linearly related to the responses of A7 animals to seven days of re-oxygenation (i.e. 13R7/13A7). Finally, panel D in Figure 4 shows 16 distinctive expression clusters of the 75 genes of the outlier group. Some of these represent immediate anoxia-induced responses with no effects at 13A7 (i.e. clusters 3 and 15) whereas another is restricted to a re-oxygenation effect (cluster 16). The gene list corresponding to these clusters is presented in the Table S2. They contain a number of muscle-relevant genes, including those involved in metabolism (e.g. enolase 3, aldolase c, GADPH), gene transcription (metal-regulated transcription factor), muscle structure and function (voltage-dependent Ca^2+^ channels, myosin light polypeptides 2 and 9, α-tubulin M, mitochondrial solute carrier protein), regulatory processes (calmodulin 3a, cell death activator) and stress responses (α-crystalline related heat shock protein).

### Functional interpretation of gene responses

**Figure 5:**
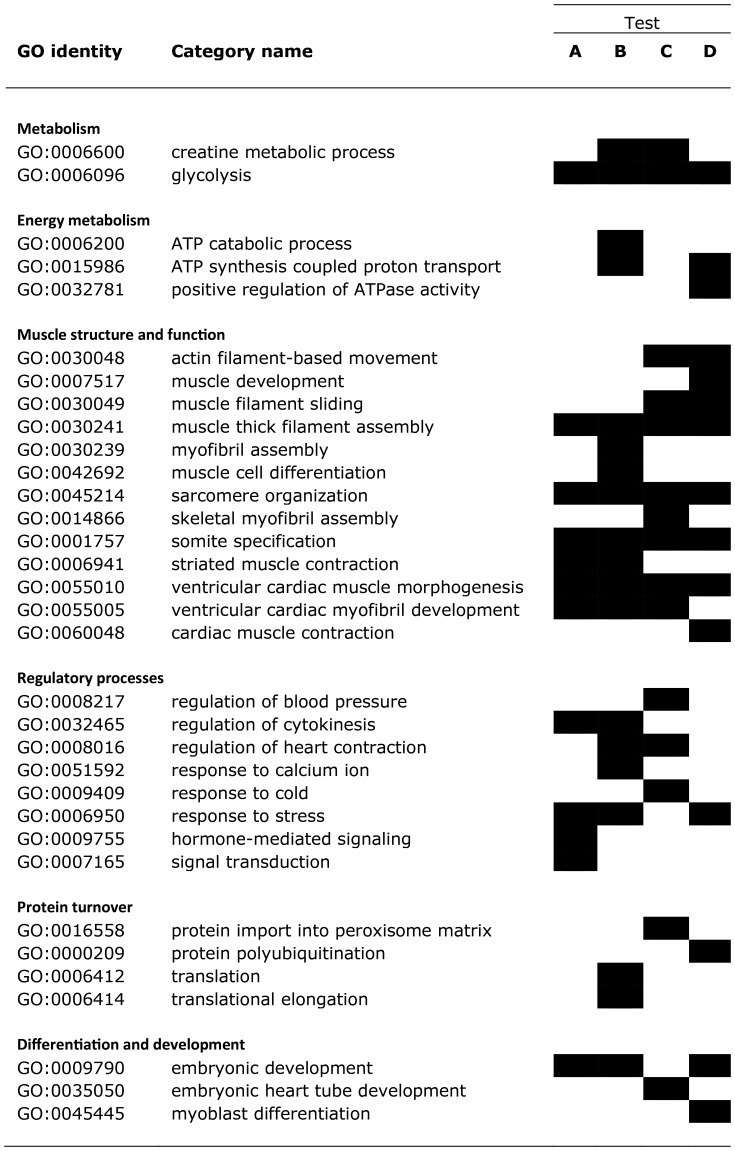
List of gene ontology (GO) categories judged as being (i) enriched in differentially expressed (DE, FDR <5%) genes according to the hypergeometric test, or (ii) displaying a significant non-random order in the *p*-rank test. The contrasts for the former test relate to all DE genes (A) or all down-regulated DE genes (B). For the *p*-rank test they relate to contrasts between temperatures under anoxia (13A7/8A7, C) and for anoxia treatment (13A7/13N7, D). GO terms have been grouped into the indicated high-level categories, which are not linked to formal GO categories.

We applied GO over-representation analysis to the complete DE gene list combining the up- and down-regulated DE groups (702 genes), and those categories deemed significant were gathered into broad functional groups (Figure 5). The rank order test explored two specific contrasts, a temperature contrast between 8 and 13°C (13A7/8A7) and a chronic anoxia contrast (13A7/13N7). We found no significant terms for the up-regulated DE genes with this test. However, by combining the GO and rank order tests, we identified several GO categories with a significant up-representation of genes, including several involved in intermediary and energy metabolism as well as protein turnover. A large number of significant categories were linked to muscle structure and function, to regulatory processes and to differentiation and development.

**Figure 6:**
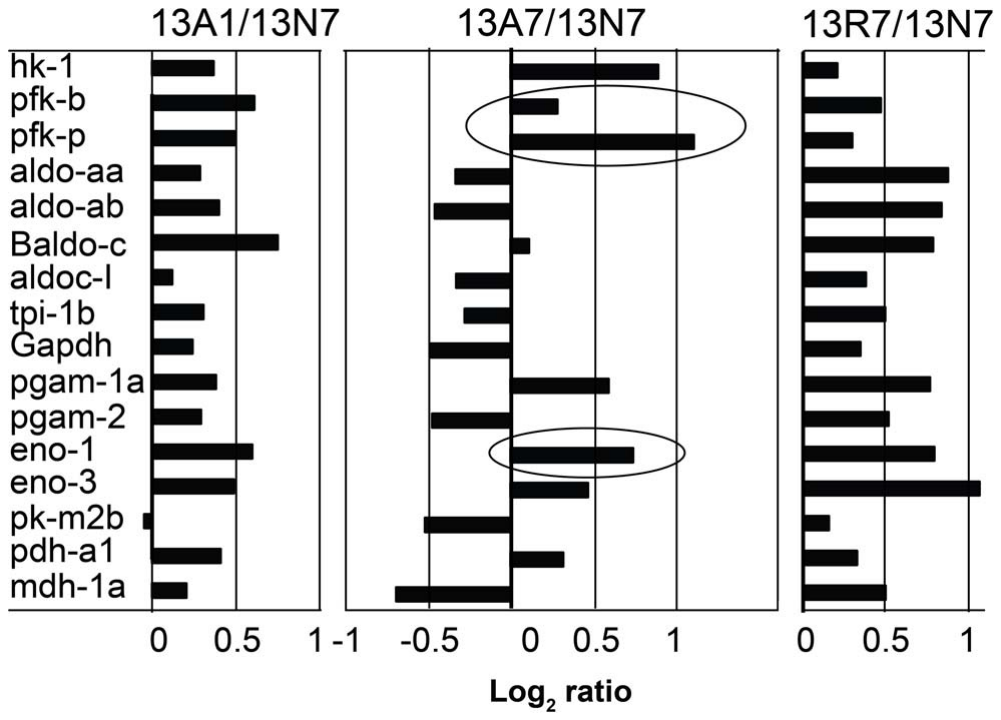
Bar-charts to illustrate the responses of genes of the glycolytic pathway to anoxia for 1 (A1/N7 log_2_ ratio) and 7 days (A7/N7), followed by re-oxygenation for a further 7 days (R7/N7). Representative gene probes were selected from all probes that aligned by BLASTx to the indicated gene identity irrespective of significance in the array analysis, as indicated by FDR <5%. The circles indicate features discussed in the text. Gene abbreviations: hk - hexokinase; pfk - phosphofructokinase, muscle b or platelet p isoforms; aldo or Baldo - aldolase with multiple isoforms; tpi - triosephosphate isomerase; gapdh - glyceraldehyde-3-phosphate dehydrogenase; pgam - phosphoglycerate mutase; eno - enolase; pk-m - pyruvate kinase-muscle; pdh - pyruvate dehydrogenase; mdh - malate dehydrogenase Isoforms are indicated.

Responses to anoxia and reoxygenation by genes of energy metabolism were of particular interest. Figure 6 displays the consensus responses of representative array probes for most of the glycolytic genes, all of which displayed an increased abundance of transcripts after 24 hours of anoxia (13A1/13N7). Of these, some (hexokinase, two isoforms of both phosphofructokinase, aldolase c and two isoforms of enolase) displayed further increases after seven days of anoxia (13A7/13N7). Others, by contrast, (three isoforms of aldolase, glyceraldehyde dehydrogenase, one isoform of phosphoglycerate mutase, two isoforms of malate dehydrogenase and the one isoform of triose phosphate isomerase) showed a decreased expression after seven days of anoxia. All genes displayed a subsequent increase on the final treatment of re-oxygenation for seven days (13R7/N7). Within these data we noted a contrasting response between isoforms of phosphoglycerate mutase-1a and -2, which is consistent with isoform switching.

Similarly, a large number of genes of mitochondrial function and the MPTP responded to seven days anoxia and re-oxygenation, including a voltage-dependent anion channel, a range of mitochondrial solute carriers for adenine and phosphate, and carriers for neurotransmitters, amino acids and creatine. Intermediary and energy metabolism categories that responded included genes for five components of the mitochondrial Fo/F1 H+ ATP synthase complex, a COX16 homolog, two muscle and two mitochondrial creatine kinase genes and two malate dehydrogenase genes.

Table S2 lists all DE genes for which a BLAST identity was established. Most of the broad functional categories were linked to muscle and the functional category includes a large number of myofibrillar proteins (α- and β-actins, cardiac myosin binding protein C, myosin light and heavy polypeptides, slow heavy myosin chain 3, titin-like protein) and Ca^2+^ regulatory proteins (a-tropomyosin and tropomyosin 4, troponin Ca^2+^ and Na^+^/K^+^ ATPase's, ryanodine receptors). The protein turnover category included 11 ribosomal proteins, two proteosomal proteins, two elongation factors and two eukaryotic translation elongation factors, as well as a range of ubiquitin-related genes. We also identified large numbers of regulatory genes, many of them linked to intracellular control *via* protein kinases and phosphatases, Ca^2+^ regulation (five calmodulin genes and calsequestrine), G proteins and GTPases. Others were linked to regulation of nuclear events, such as transcription factors, high mobility group proteins and histone modifying enzymes, ring finger proteins, Y-box binding proteins and RNA polymerases. Interestingly, a range of genes in the systems-level regulation category included receptors for aryl hydrocarbon, oestrogen, oxysterol, GABA, TNF, FGF, adenosine and insulin, as well as transducers of ERBB2 and glycoprotein hormones. The stress-related category of genes included two cold-inducible RNA binding proteins, hypoxia up-regulated protein, four heat shock proteins and two chaperones, and two genes involved in oxygen metabolism.

### Ingenuity pathway analysis

Using the DE list of the anoxia response genes without outlier genes (702 genes), we then searched the IPA knowledge database for evidence for coherent gene responses across a range of canonical functional pathways. Two canonical pathways are presented with significant up regulation in red and down regulation in green. Figure S1 shows Ca^2+^ handling, with significant anoxia-induced reduction of transcripts for the voltage-gated calcium channel, protein phosphatase 1, which is an inhibitor of the Ca^2+^ release-activated Ca^2+^ channel. We also saw a reduction in calmodulin (CALM) and in ryanodine receptors (RYR), and increases for the plasma membrane Ca^2+^ ATPase (PMCA) and calcium-transporting ATPase type 2C member 1 (ATP2C1).

Figure S2 illustrates genes connected to NF-kB, a protein complex that controls transcriptions of DNA and which is involved in stress responses. Up-regulated genes included TNF receptor-associated factor 1 (TRAF1), Nucleotide-binding oligomerization domain-Like Receptor with a Pyrin domain 3 (NLRP3), NF-kappa-B inhibitor zeta (NFKBIZ) while guanine nucleotide-binding protein subunit beta (GNB1) were down-regulated. Transcripts from genes linked to NFkB pathways were also significantly changed, including an increase in connective tissue growth factor (CTGF) and fibroblast growth factor 1 (FGF1).

### Validation of array data by comparison with RT-PCR

We have compared the fold-change values determined by the microarray method with those of RT-PCR. For this, we selected three genes and determined fold-change values for three contrasts (13A1/13N7, 13A7/13A1 and 13A7/13R7). In all cases, values were normalised using the corresponding values for β-actin (Ellefsen et al 2008). For each gene we observed an orderly, linear relationship between the two techniques, with a slope approximating unity (Figure S3).

## Discussion

Living without oxygen represents such a large physiological challenge that we expected substantial changes in the transcriptome in the crucian carp heart. Indeed, based on a FDR of <5% we identified 777 unique genes that showed changes in transcript abundance between normoxia and anoxia at 13°C, and a near complete reversal following an additional seven days under normoxia. Anoxia responses required up to seven days of exposure to develop completely and we found limited evidence of early responses within the first 24 hours of anoxic exposure. By contrast exposure to anoxia at 8°C revealed very few responding genes at FDR <5%. Given previous work demonstrating strong transcriptional responses in the related common carp, *C. carpio*, subjected to equivalent reductions in temperature [28], we found surprisingly few genes that differ between the 13°C- and 8°C-acclimated crucian carp at any of the anoxia/reoxygenation sampling time points though the 7-day anoxia (8A7 vs 13A7) group displayed 67 responding unique genes.

However, we point to two potentially confounding factors in concluding that no responses were evident at 8°C. First, long-term acclimation at 13°C and 8°C were chosen to represent early and late phases, respectively, of the normal seasonally-entrained temperature change as these fish enter the winter months when anoxia can be anticipated in the natural habitat [46]. Thus, by being thermal acclimations they do not strictly relate to acute responses to temperature, as revealed in the common carp study [28]. Although both 8 and 13°C groups were held on a 12 hours light/12 hours dark cycle, we cannot exclude more subtle seasonally entrained influences, such as those linked to photoperiod, reproductive cycles or reduced appetite etc., which might modify responses. Secondly, we found that the fold-change values of the differentially expressed, anoxia-responsive genes at 13°C were positively correlated to smaller anoxia-induced changes in expression of the same genes at 8°C (Figure S4). This is despite the fact that gene responses at 8°C lay beneath the 5% FDR criterion that we employed. It follows that the main features of the transcriptomic response to anoxia were conserved at both temperatures, but the magnitude of response was temperature-dependent either due to direct thermal effects or perhaps due to the greater energetic demand of tissues at 13 compared to 8°C.

### Pathway and Process Analysis

#### Glycolysis

The GO enrichment analysis indicated that several pathways and processes were regulated by the anoxia treatment at 13°C, including glycolysis, which is the only ATP generating pathway able to support sustained cardiac activity during chronic anoxia [7], [20], [47]. The regulation of glycolytic flux is widely thought to be dependent upon three key regulatory enzymes, namely hexokinase, phosphofructokinase and pyruvate kinase, while the remaining steps comprise equilibrium enzymes whose expression does not greatly influence glycolytic flux. A deeper analysis of our transcript data revealed some complexity in the responses of glycolytic genes at 13°C. First, all glycolytic genes displayed a small but consistently up-regulated transcript abundance relative to the control after one day of anoxia, and after seven days of reoxygenation. However, responses following the intervening seven days of anoxia varied; five genes were up-regulated, including hexokinase and phosphofructokinase [48], and two isoforms of enolase, which is an equilibrium enzyme. By contrast, all other glycolytic genes assessed were down-regulated, among which only one, pyruvate kinase (*pk-m2b*), was regulatory.

The induction during anoxia of the two regulatory steps is consistent with the widely held expectation of up-regulated glycolysis during anoxia [2]. Hexokinase, is the first enzyme of glycolysis, and its activity is inhibited by its own product, glucose-6-phosphate [48]. Anoxic up-regulation may thus be responsible for ensuring a constant flow of glucose into the cell given that glucose-6-phosphate is not easily transported across cell membranes [49]. Phosphofructokinase is allosterically inhibited by ATP, the first irreversible step in “trapping” glucose in this pathway [48]. But crucian carp heart maintains the cellular ATP concentrations constant during anoxia [20]. However, it would be reasonable to speculate that local changes in ATP levels within cells will occur due to lack of oxidative phosphorylation. The last important regulatory point in glycolysis involves pyruvate kinase. The regulatory residues of pyruvate kinase are highly conserved [50] and, as with phosphofructokinase, is allosterically inhibited by ATP [51]. Again intracellular shifts in ATP levels may allow for an increase in pyruvate kinase activity due to a locally low ATP in the vicinity of the enzyme.

Taken together, we speculate that up-regulation of key transcripts in glycolysis results in a sustained, or even increased glycolytic flux in anoxia, despite the small changes in the majority of transcripts.

#### Other glycolytic intermediates

Surviving anoxia with a glycolytic energy supply likely involves the prevention of glycolytic intermediates from glycating proteins and other macromolecules [52]. One side-product of glycolysis is methylglyoxal (MG), which is created mainly from the triosephosphate intermediates, glyceraldehyde-3-phosphate and dihydroxyacetone phosphate [53]. MG is associated with oxidative stress and aging [53] and scavenging of MG attenuates ischemia-reperfusion injury in cultured cardiomyocytes [54]. One of the important systems in handling MG is the glyoxalase system composed of glyoxalase I and glyoxalase II [55]. In the anoxic crucian carp, we found a rapid (within 24 hours) transcriptional up-regulation of glyoxalase I in all anoxic conditions, this being one of the few immediate-early transcriptomic responses to anoxia (see Table S1). Likewise, we found a transcriptional up-regulation of malic enzyme, which catalyses the conversion of malate to pyruvate with the concomitant reduction of NAD(P)+ to NAD(P)H. In contrast to most mammals, where malic enzyme utilises NADP+, fishes have two subtypes of malic enzyme, one preferring NAD+, the other preferring NADP+ [56]. This could indicate a two-fold action of malic enzyme in anoxic crucian carp, one by converting pyruvate into malate, regenerating NAD+ for glycolysis, the other converting malate into pyruvate, creating NADPH for handling oxidative stress. We also found transaldolase to be transcriptionally up-regulated in our data consistent with activation of the pentose phosphate pathway (PPP). This pathway is important for generating the cellular requirement for NADPH [57], which is an important cofactor that can protect against oxidative stress by maintaining the redox potential of the glutathione:glutathione disulphide (GSH/GSSG) ratio [58].

#### Mitochondrial function

Another finding from the GO analysis relates to the regulation of mitochondrial function. In mammals, a central driver of mitochondrial dysfunction during ischemia-reperfusion injury is the formation of the mitochondrial permeability transition pore (MPTP) [9], [13]. Opening of the MPTP is followed by myocyte death due to collapse of the mitochondrial membrane potential and release of cytotoxic elements into the cytosol [13]. The current view in mammals is that the MPTP is formed of a heterodimer between adenine nucleotide translocase (ANT) and mitochondrial phosphate carrier (16), which on binding with cyclophyline-D forms a complex that together with increased matrix [Ca^2+^] and increased pH causes the opening of the MPTP channel. Voltage-dependent anion channel (VDAC) and hexokinase have been shown to have a regulatory role in MPTP opening [13]. Interestingly, we found anoxia-induced changes in transcript abundance of all of these proteins except cyclophyline-D; ANT and VDAC were reduced two-fold in anoxia while hexokinase increased 2-fold. We also found an up-regulation of mitochondrial phosphate carrier in the anoxic heart. The varied responsiveness of MPTP-related transcripts suggests regulation of the pore in anoxia.

GO analysis also suggests changes in the potential H^+^-coupled ATP synthesis. We found that 5 components of the ATP-producing Fo/F1 H^+^ ATPase were transcriptionally reduced, suggesting an uncoupling of the ATP-producing steps. In addition, uncoupling protein 1 was up-regulated. Mitochondrial function in anoxia is not known, but O_2_ cannot be the main electron acceptor. From this it can be deduced that the proton gradient might be jeopardised, and as a consequence the Fo/F1 ATPase would be down-regulated. One hypothesis might be that the ATPase works in reverse mode to prevent mitochondrial depolarization as it does in mammalian tissues in early ischemia [16]. However, during anoxia this represents a waste of ATP, at a time when ATP provisioning is the key factor in survival. A closer exploration of mitochondrial function in anoxia is clearly needed.

#### Ca^2+^ handling

Many aspects of Ca^2+^ handling were altered by anoxia. At the cell membrane, transcripts for the voltage-gated calcium channel were reduced, while those for plasma membrane Ca^2+^ ATPase were increased. In addition, the protein phosphatase 1 transcript was increased, which may function to inhibit Ca^2+^ release-activated Ca^2+^ channel. These changes are consistent with a reduced flux of Ca^2+^ across the myocyte membrane. Moreover, the ATP2C1 was transcriptionally up-regulated indicating that Ca^2+^ could be removed from the cytosol faster (Figure S1). Together these effects indicate a reduced Ca flux in crucian carp myocytes.

#### NF-kB

We found several significant responses in genes linked to NFkB signalling (see Figure S2). NFkB has been reported to act as an oxygen-responsive transcription factor in anoxia-tolerant turtles [59]. NFkB also initiates the immune response by targeting several cell signalling events, finally altering the transcription of several genes [60]. It is also linked to both cardiovascular health and disease by regulating responses to stress, hypoxia and ischemia [61]. We found an up-regulation of TRAF1, which is essential for mediating TNFα-mediated activation of NFkB [62]. NLRP3 was upregulated, indicating activation of the innate immune system [63].

### Perspective

Our discovery of 777 genes that display transcriptional regulation during anoxia offers a number of new hypotheses for exploring how cardiac performance is sustained in the crucian carp during long-term anoxic exposure. This includes anoxia-induced regulatory processes operating in key pathways, notably in energy provisioning, mitochondrial performance, handling of Ca^2+^, and intracellular signalling. Because transcriptional regulation is just one element within many, these outcomes need to be verified and extended in broader studies of each process. Importantly, these predominant anoxia-induced transcriptional responses were entirely reversed when normoxia had been re-established, revealing reversibility and indicating that the changes seen in anoxia are of adaptive and protective nature.

## Supporting Information

Figure S1 **Regulated transcripts related to Ca^2+^ handling generated by Ingenuity.** Up-regulation of a gene is indicated by a black colour of a corresponding block. Down-regulated functions are marked in grey. White blocks correspond to functions that were missing from the input list of differentially expressed genes. The following transcripts were significantly regulated: VGCC (Voltage dependent Ca^2+^ channel), CALM (Calmodulin), RyR (Ryanodine receptor), PP1 (Protein phosphatase 1), Aralar (Ca ^2+^ sensitive shuttle), PMCA (Plasma membrane Ca ^2+^ ATPase), ATP2C1 (Ca^2+^ transporter type 2C1), TropT (Troponin T), Mybp3 (Cardiac myosine binding protein 3), Tropomyosin, Actin-α.
(TIF)

Figure S2 **Regulated transcripts related to NF-kB signalling generated by Ingenuity.** Up-regulation of a gene is indicated by a black colour of a corresponding block. Down-regulated functions are marked in grey. White blocks correspond to functions that were missing from the input list of differentially expressed genes. Dashed lines in diagrams correspond to physical interactions and solid lines to regulatory interactions between gene products. The following transcripts were significantly regulated in anoxic signalling via NFkB in crucian carp hearts: HCCS (Holocytochrome-c synthase), SMC6 (Structural maintenance of chromosome protein 6), MAPK6 (Mitogen-activated protein kinase 6), XIAP (X-linked inhibitor of apoptosis protein), NFKBIZ (Nuclear factor kappa beta inhibitor zeta, CGA (Glycoprotein hormone alpha), PIM3 (Protein kinase, pim-3 oncogen), HSPB1 (Heat shock protein 1, HSP27 protein 1), HSP27 (Heat shock protein 27), HSPB7 (Heat shock protein 27, member 7), NLRP3 (Nod-like receptor pyrine domain 3), THBS1 (Thrombospondin 1), MAPKAPK3 (Mitogen activated protein kinase kinase 3), GNB1 (GTB-binding regulatory protein beta 1), SLC25A3 (Mitochondrial phosphate carrier), SLC25A4 (Mitochondrial ADP/ATP translocase 1, ANT1), SLC25A5 (Mitochondrial ADP/ATP translocase 5), SLC11A2 (proton coupled divalent metal ion transporter member 2), TRAF1 (TNF receptor-associated factor 1), CTGF (connective tissue growth factor), FGF1 (Fibroblast growth factor 1), JUND (Jun-D proto-ocogen), Sapk (Stress activated protein kinase), DUSP8 (Dual specificity phosphatase 8), MAPK12 (Mitogen activated protein kinase 12, ERK3), VDAC2 (Voltage dependent anion channel 2) and SPOP (speckle-type POZ protein). The Figure also indicates connections to known cardiovascular physiology or disease stated.
(TIF)

Figure S3 **Validation of differentially expressed genes.** The relationship of the log_2_ fold-change values generated by the microarray technique with that generated by RT-PCR. We tested 3 genes (*gapdh*, *ppia* and *hsc70*). For all three, the log_2_ fold-change values represent comparisons between A7/N7, A1/N7 and R7/N7/. PCR results were normalised to expression of β-actin acting as loading control. The slope of the best-fit line with regression constants is indicated for each gene.
(TIF)

Figure S4
**Comparison of anoxia-induced gene expression at 8 with 13°C.** The expression values for the A7/N7 ratio for the 8°C experiment plotted against that for that of the 13°C experiment. The heavy solid line represents the line of regression with a slope of 0.17. The light line indicates equality between the two datasets, slope  = 1. The correlation coefficient between the responses on day 7 at 13°C (13A7/13N7) and at 8°C (8A7/8N7) was r = 0.37, and the length of the response data vector is 844. Substituting *r* = 0.37 and *n* = 844 into *t*-transformation 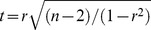, we obtained a sample value *t* = 11.73 being equivalent to a random draw from a *t* distribution of 842 degree freedom. A two-sided test of this t value yields a very small p-value 1.46E-29 which confirms that the two responses were positively and very significantly correlated.(TIF)

Table S1 **Details of the responses of the microarray probe that BLASTx's to Glyoxalase, to 1, and 7 days of anoxia treatment of crucian carp, followed by 7 days of re-oxygenation (A1, A7 and R7, respectively).** All values are expressed as log2 ratio relative to the control, untreated condition (i.e., N7). Also provided are the p and q values for each contrast.
(DOCX)

Table S2 **List of 291 genes selected from the 777 judged as being differentially expressed in at least one of the indicated contrasts in the experimental design in** **Fig 1** **, together with those responding to temperature.** The genes have been collated into broad functional groups. The ‘cluster’ column indicates to which cluster in Fig 4 of the accompanying paper each gene belonged – U indicates anoxia up-regulated genes, D- anoxia downregulated genes in Figure 4B while cluster 1–16 indicates to which outlier group in Fig 4D. T- indicates temperature-regulated genes not shown in Fig 4. We show the log_2_ ratio for two contrasts, as indicated. Not listed are genes lacking a meaningful functional annotation, and others from other functional categories.
(DOCX)
