# Supplementary figures and images for: Life without Oxygen: Gene Regulatory Responses of the Crucian Carp (Carassius carassius) Heart Subjected to Chronic Anoxia
Source: PLoS One. 2014 Nov 5;9(11):e109978. doi: 10.1371/journal.pone.0109978 (PMC4220927; doi:10.1371/journal.pone.0109978)

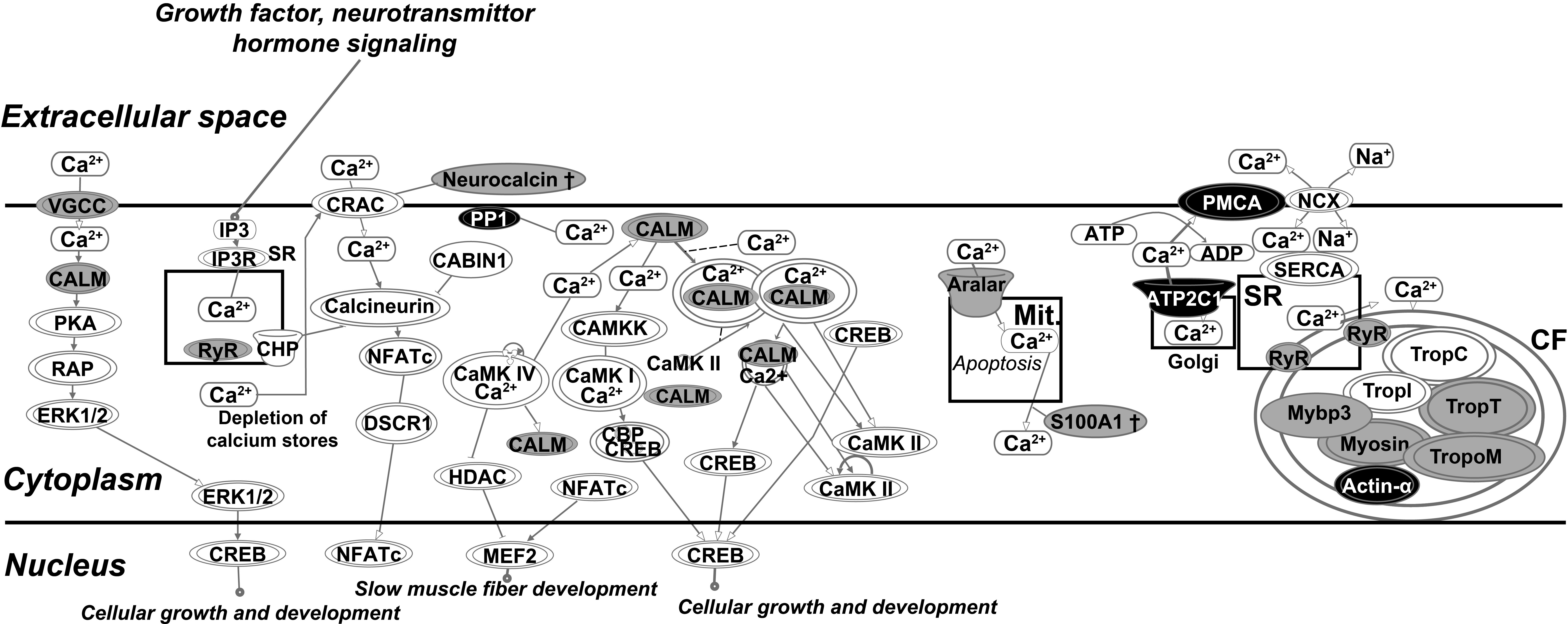

Supplement: Figure S1 — Regulated transcripts related to Ca2+ handling generated by Ingenuity. Up-regulation of a gene is indicated by a black colour of a corresponding block. Down-regulated functions are marked in grey. White blocks correspond to functions that were missing from the input list of differentially expressed genes. The following transcripts were significantly regulated: VGCC (Voltage dependent Ca2+ channel), CALM (Calmodulin), RyR (Ryanodine receptor), PP1 (Protein phosphatase 1), Aralar (Ca 2+ sensitive shuttle), PMCA (Plasma membrane Ca 2+ ATPase), ATP2C1 (Ca2+ transporter type 2C1), TropT (Troponin T), Mybp3 (Cardiac myosine binding protein 3), Tropomyosin, Actin-α. (TIF) [file pone.0109978.s001.tif]

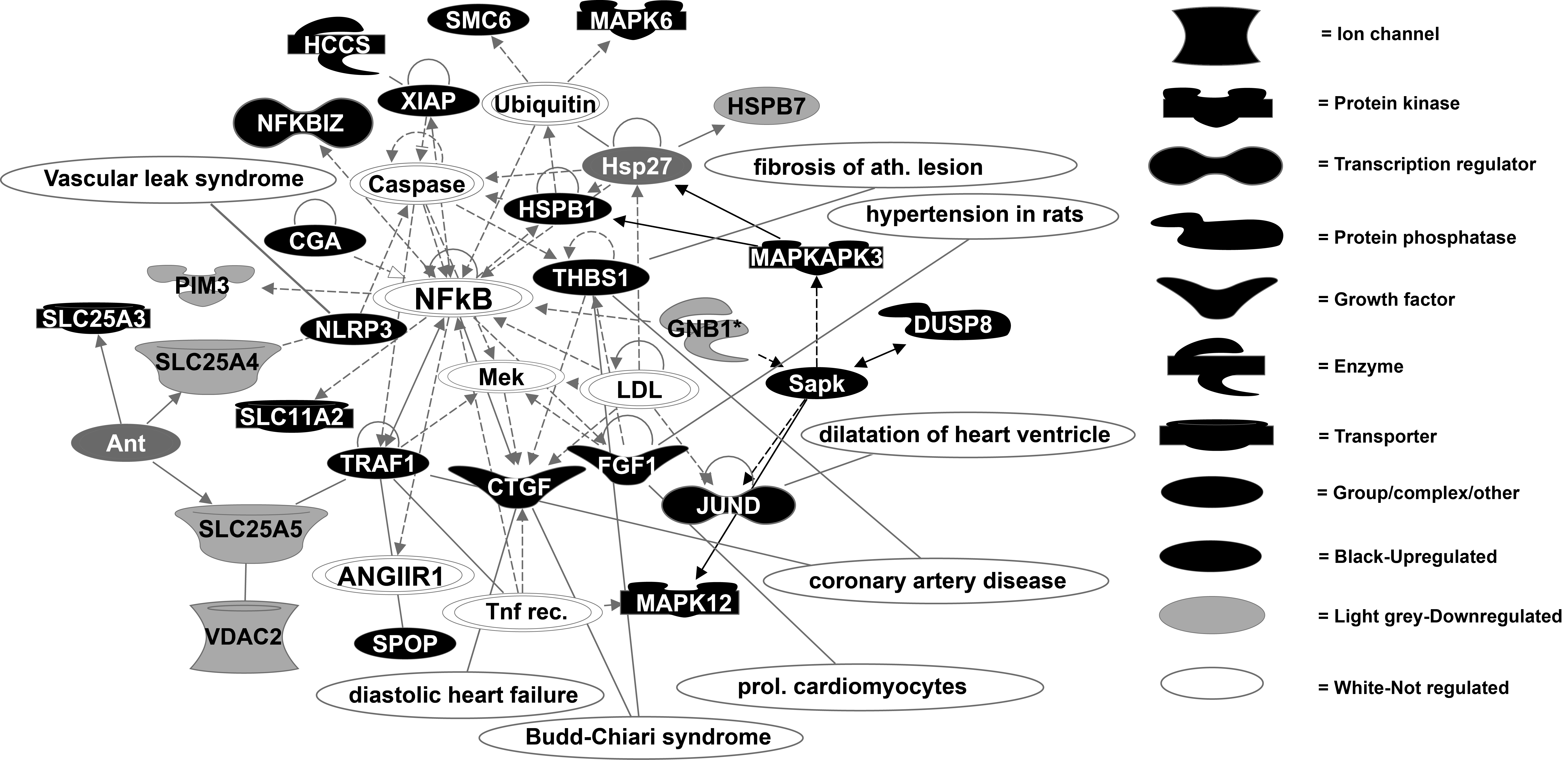

Supplement: Figure S2 — Regulated transcripts related to NF-kB signalling generated by Ingenuity. Up-regulation of a gene is indicated by a black colour of a corresponding block. Down-regulated functions are marked in grey. White blocks correspond to functions that were missing from the input list of differentially expressed genes. Dashed lines in diagrams correspond to physical interactions and solid lines to regulatory interactions between gene products. The following transcripts were significantly regulated in anoxic signalling via NFkB in crucian carp hearts: HCCS (Holocytochrome-c synthase), SMC6 (Structural maintenance of chromosome protein 6), MAPK6 (Mitogen-activated protein kinase 6), XIAP (X-linked inhibitor of apoptosis protein), NFKBIZ (Nuclear factor kappa beta inhibitor zeta, CGA (Glycoprotein hormone alpha), PIM3 (Protein kinase, pim-3 oncogen), HSPB1 (Heat shock protein 1, HSP27 protein 1), HSP27 (Heat shock protein 27), HSPB7 (Heat shock protein 27, member 7), NLRP3 (Nod-like receptor pyrine domain 3), THBS1 (Thrombospondin 1), MAPKAPK3 (Mitogen activated protein kinase kinase 3), GNB1 (GTB-binding regulatory protein beta 1), SLC25A3 (Mitochondrial phosphate carrier), SLC25A4 (Mitochondrial ADP/ATP translocase 1, ANT1), SLC25A5 (Mitochondrial ADP/ATP translocase 5), SLC11A2 (proton coupled divalent metal ion transporter member 2), TRAF1 (TNF receptor-associated factor 1), CTGF (connective tissue growth factor), FGF1 (Fibroblast growth factor 1), JUND (Jun-D proto-ocogen), Sapk (Stress activated protein kinase), DUSP8 (Dual specificity phosphatase 8), MAPK12 (Mitogen activated protein kinase 12, ERK3), VDAC2 (Voltage dependent anion channel 2) and SPOP (speckle-type POZ protein). The Figure also indicates connections to known cardiovascular physiology or disease stated. (TIF) [file pone.0109978.s002.tif]

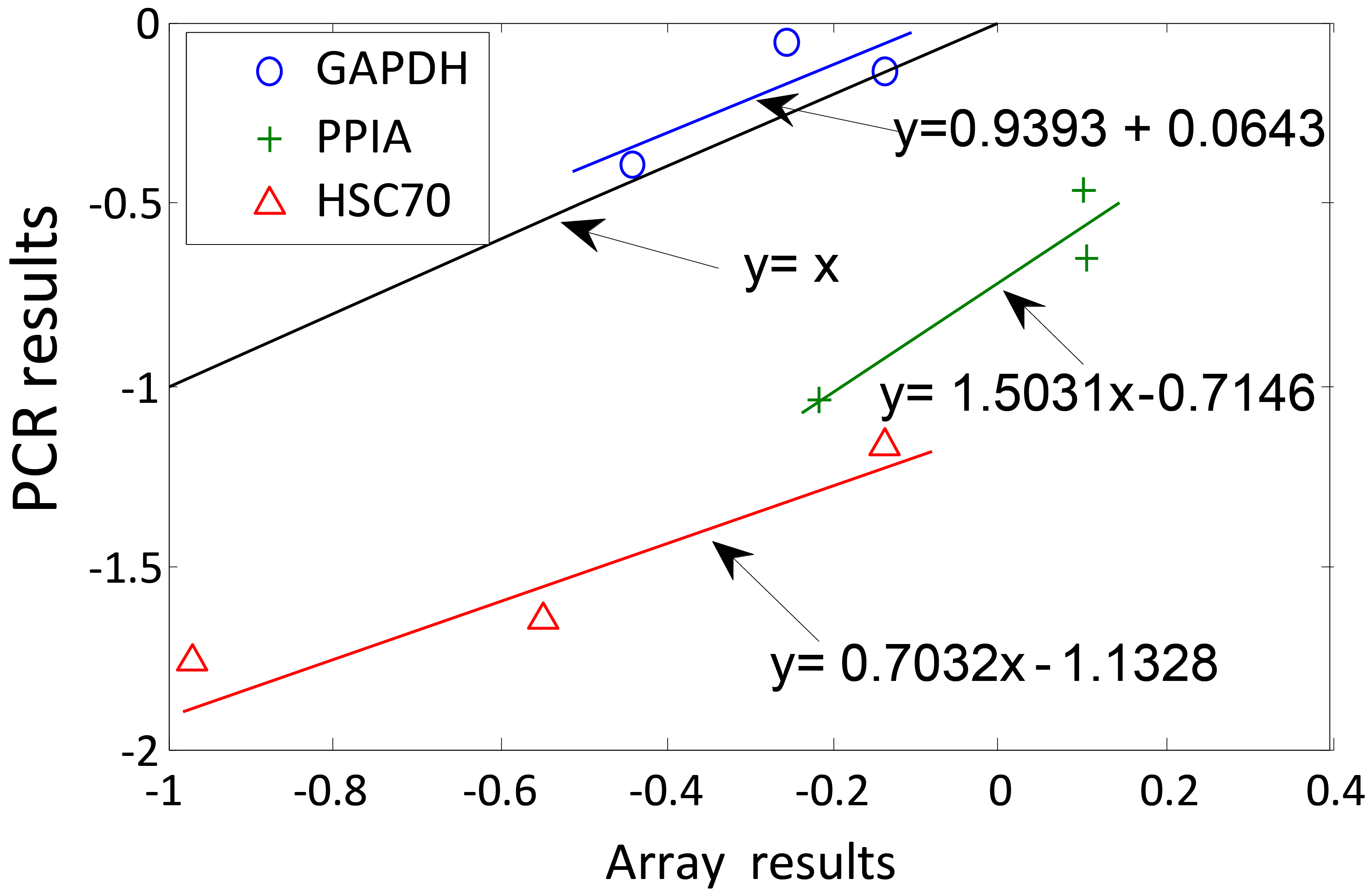

Supplement: Figure S3 — Validation of differentially expressed genes. The relationship of the log2 fold-change values generated by the microarray technique with that generated by RT-PCR. We tested 3 genes (gapdh, ppia and hsc70). For all three, the log2 fold-change values represent comparisons between A7/N7, A1/N7 and R7/N7/. PCR results were normalised to expression of β-actin acting as loading control. The slope of the best-fit line with regression constants is indicated for each gene. (TIF) [file pone.0109978.s003.tif]

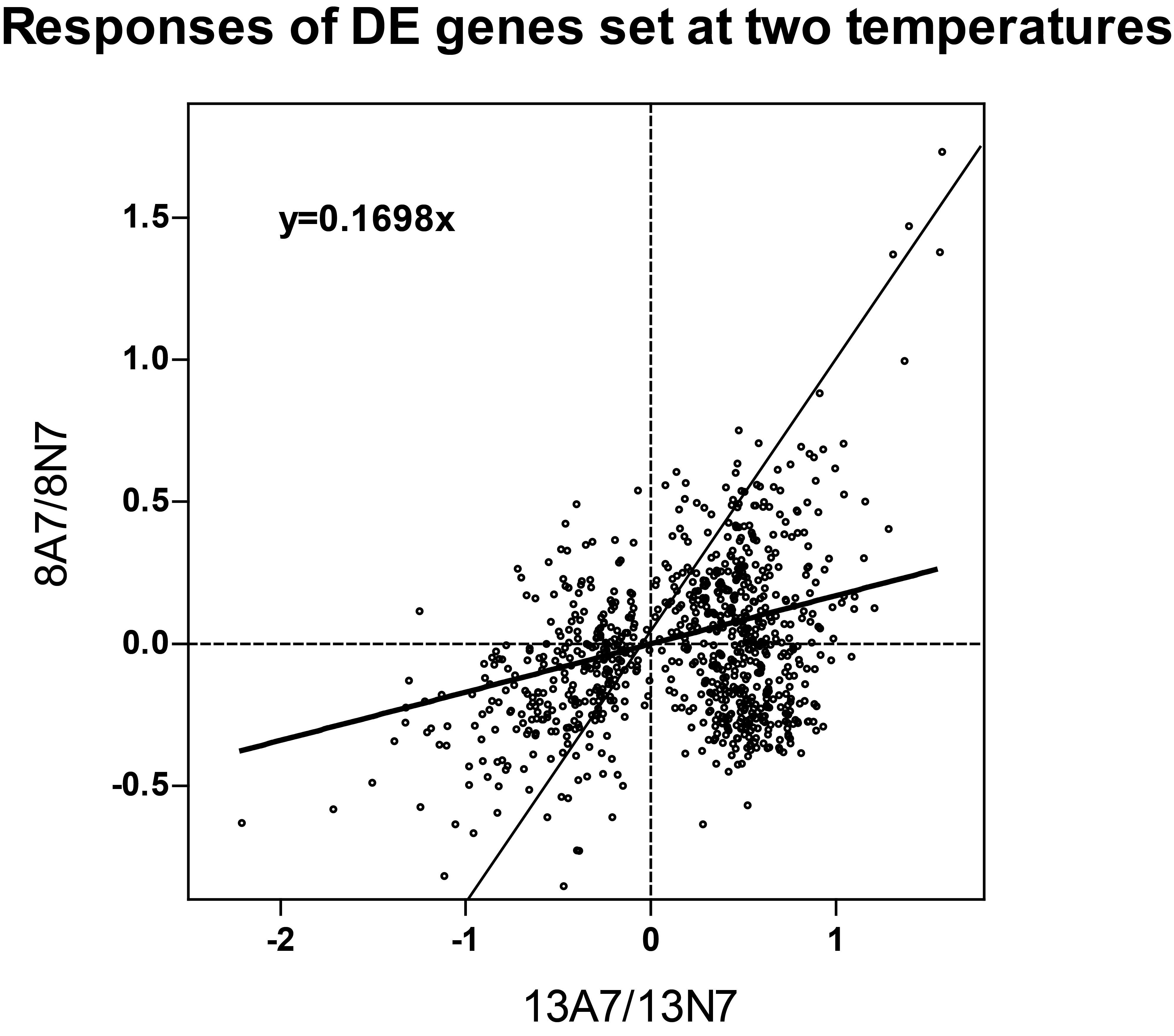

Supplement: Figure S4 — Comparison of anoxia-induced gene expression at 8 with 13°C. The expression values for the A7/N7 ratio for the 8°C experiment plotted against that for that of the 13°C experiment. The heavy solid line represents the line of regression with a slope of 0.17. The light line indicates equality between the two datasets, slope = 1. The correlation coefficient between the responses on day 7 at 13°C (13A7/13N7) and at 8°C (8A7/8N7) was r = 0.37, and the length of the response data vector is 844. Substituting r = 0.37 and n = 844 into t-transformation , we obtained a sample value t = 11.73 being equivalent to a random draw from a t distribution of 842 degree freedom. A two-sided test of this t value yields a very small p-value 1.46E-29 which confirms that the two responses were positively and very significantly correlated. (TIF) [file pone.0109978.s004.tif]
