# Supplementary material for: Life without Oxygen: Gene Regulatory Responses of the Crucian Carp (Carassius carassius) Heart Subjected to Chronic Anoxia
Source: PLoS One. 2014 Nov 5;9(11):e109978. doi: 10.1371/journal.pone.0109978 (PMC4220927; doi:10.1371/journal.pone.0109978)
Supplement: Table S1 — Details of the responses of the microarray probe that BLASTx's to Glyoxalase, to 1, and 7 days of anoxia treatment of crucian carp, followed by 7 days of re-oxygenation (A1, A7 and R7, respectively). All values are expressed as log2 ratio relative to the control, untreated condition (i.e., N7). Also provided are the p and q values for each contrast. (DOCX) [file pone.0109978.s005.docx]

**Supplementary Table S2**

| **Gene ID** | **Genename** | **Cluster** | | **13A7/13N** | **13R7/13N** | |
| --- | --- | --- | --- | --- | --- | --- |
|  |  |  |  | |  |  |
| **Cytoplasm** |  |  |  | |  |  |
| 83e18 | ankyrin and armadillo repeat containing (ankar) transcript variant 2 | U | 0.513 | | -0.235 |  |
| 53b16 | centrosomal protein 76 (cep76) | U | 0.182 | | -0.112 |  |
| 24h08 | clusterin (complement lysis inhibitor SP-4040 sulfated glycoprotein 2 testosterone-repressed prostate message 2 apolipoprotein J) (clu) | D | -0.094 | | 0.091 |  |
| 62i24 | dynamin 1-like (dnm1l) | U | 0.500 | | -0.346 |  |
| 58m03 | dynamin 2 (dnm2) | U | 0.637 | | -0.126 |  |
| 25d11 | similar to alpha-tubulin isotype M-alpha-2 (MGC171407) | D | -0.136 | | 0.020 |  |
| 54d24 | PREDICTED: similar to ankyrin 3 (LOC794638) | U | 0.379 | | -0.579 |  |
| 51l08 | PREDICTED: similar to ankyrin repeat and SOCS box-containing 14 (LOC566775) | D | -0.314 | | 0.810 |  |
| 66e10 | PREDICTED: similar to dedicator of cytokinesis 8 (Dock8) | 7 | 1.085 | | -0.316 |  |
| 54b02 | PREDICTED: similar to desmin (LOC567459) | U | 0.415 | | -0.582 |  |
| 25c16 | PREDICTED: similar to microfibrillar-associated protein 4 (LOC100007488) | U | 0.059 | | -0.402 |  |
| 62b04 | PREDICTED: similar to microfibrillar-associated protein 4 (LOC100007560) | U | 0.643 | | -0.245 |  |
| 65p21 | PREDICTED: similar to microtubule associated serine/threonine kinase 3 (LOC559371) | U | 0.897 | | -0.338 |  |
| 77o01 | PREDICTED: similar to microtubule associated serine/threonine kinase family member 4 (LOC795151) | 16 | 0.189 | | -0.810 |  |
| 53b06 | PREDICTED: similar to microtubule-associated protein 1 A (LOC797545) | U | 0.362 | | -0.358 |  |
| 52o18 | PREDICTED: similar to protocadherin 2A3 (LOC100000418) | U | 0.743 | | -0.166 |  |
|  |  |  |  | |  |  |
| **Differentiation and development** | |  |  | |  |  |
| 14o02 | egl nine homolog 1 (C. elegans) (egln1) | 15 | 1.309 | | -0.018 |  |
| 83f04 | homeo box C6a (hoxc6a) | D | -0.258 | | 0.104 |  |
| 79n08 | homeo box C9a (hoxc9a) | U | 0.524 | | -0.219 |  |
| 21f17 | PREDICTED: homeo box C10a (hoxc10a) | U | 0.422 | | -0.290 |  |
| 17a03 | PREDICTED: similar to hedgling (LOC562711) | T | 0.514 | | -0.114 |  |
| 75p16 | PREDICTED: similar to osteoblast differentiation promoting factor protein (LOC563198) | U | 0.199 | | -0.275 |  |
| 66n16 | PREDICTED: similar to pleckstrin homology domain containing family A member 6 (LOC568764) | U | 0.916 | | -0.429 |  |
| 54p01 | PREDICTED: similar to son of sevenless homolog 2 (LOC561670) | U | 0.740 | | -0.311 |  |
| 22d18 | vascular endothelial growth factor Ab (vegfab) | D | 1.562 | | -0.321 |  |
| 40h11 | betacellulin epidermal growth factor family member (btc) | D | -0.219 | | 0.038 |  |
|  |  |  |  | |  |  |
| **Intermediary and energy metabolism** | |  |  | |  |  |
| 26e05 | creatine kinase brain b (ckbb) | D | -0.515 | | 0.337 |  |
| 61i19 | creatine kinase mitochondrial 1 (ckmt1) nuclear gene encoding mitochondrial protein | T | -0.442 | | 0.157 |  |
| 33k07 | creatine kinase mitochondrial 2 (sarcomeric) (ckmt2) nuclear gene encoding mitochondrial protein | D | 0.031 | | 0.240 |  |
| 30o21 | creatine kinase muscle a (ckma) | D | -0.497 | | 0.143 |  |
| 66d12 | cytochrome b-245 beta polypeptide (chronic granulomatous disease) (cybb) | U | 0.904 | | -0.049 |  |
| 22f21 | holocytochrome c synthase (hccs) | U | 0.553 | | -0.139 |  |
| 14f23 | translocase of inner mitochondrial membrane 17 homolog A (yeast) (timm17a) nuclear gene encoding mitochondrial protein | U | 0.314 | | -0.102 |  |
| 82a23 | aldolase a fructose-bisphosphate a (aldoaa) | D | -0.328 | | 0.770 |  |
| 30c03 | aldolase a fructose-bisphosphate b (aldoab) | D | -0.408 | | 0.467 |  |
| 60b24 | aldolase c fructose-bisphosphate like (aldocl) | D | -0.323 | | 0.332 |  |
| 30c16 | enolase 3 (beta muscle) (eno3) | D | -0.160 | | 0.450 |  |
| 13i23 | glyceraldehyde-3-phosphate dehydrogenase (gapdh) | D | -0.581 | | 0.312 |  |
| 27i01 | phosphoglycerate kinase 1 (pgk1) | D | -0.443 | | 0.989 |  |
| 28a22 | triosephosphate isomerase 1b (tpi1b) | D | -0.272 | | 0.436 |  |
| 64l02 | 1-acylglycerol-3-phosphate O-acyltransferase 9 (agpat9) | T | 0.353 | | -0.536 |  |
| 06n21 | aminolevulinate delta- synthetase 1 (alas1) | T | 1.041 | | -0.170 |  |
| 81m19 | glutamate-ammonia ligase (glutamine synthase) a (glula) | D | -0.193 | | -0.126 |  |
| 82g22 | glutamic-oxaloacetic transaminase 1 soluble (got1) | U | 0.775 | | -0.220 |  |
| 25e03 | glutamic-oxaloacetic transaminase 2a mitochondrial (aspartate aminotransferase 2) (got2a) nuclear gene encoding mitochondrial protein | D | -0.234 | | 0.093 |  |
| 30j10 | glutamic-oxaloacetic transaminase 2b mitochondrial (aspartate aminotransferase 2) (got2b) nuclear gene encoding mitochondrial protein | D | -0.193 | | 0.097 |  |
| 20f14 | hypoxanthine phosphoribosyltransferase 1 (hprt1) | U | 0.372 | | -0.260 |  |
| 54f09 | inositol(myo)-1(or 4)-monophosphatase 1 (impa1) | U | 0.221 | | -0.308 |  |
| 78e12 | lactate dehydrogenase B4 (ldhb) | D | -0.680 | | -0.133 |  |
| 82d12 | malic enzyme 1 NADP(+)-dependent cytosolic (me1) | U | 0.586 | | -0.361 |  |
| 53m15 | methionine sulfoxide reductase B3 (msrb3) | U | 0.405 | | -0.773 |  |
| 78f24 | O-linked N-acetylglucosamine (GlcNAc) transferase (UDP-N-acetylglucosamine:polypeptide-N-acetylglucosaminyl transferase) (ogt) transcript variant 1 | 14 | 0.297 | | -0.852 |  |
| 05l11 | protein arginine methyltransferase 1 (prmt1) | D | -0.414 | | 0.216 |  |
| 34o08 | pyrroline-5-carboxylate reductase 1 (zgc:73112) | T | 0.333 | | -0.320 |  |
| 80i05 | transaldolase 1 (taldo1) | U | 0.639 | | 0.069 |  |
| 09c15 | PREDICTED: amylo-1 6-glucosidase 4-alpha-glucanotransferase (agl) | D | -0.330 | | -0.008 |  |
| 18o09 | PREDICTED: novel protein similar to vertebrate diacylglycerol kinase beta 90kDa (DGKB) (LOC567728) | U | 0.482 | | -0.274 |  |
| 73j01 | PREDICTED: similar to arylsulfatase I (LOC563760) | U | 0.369 | | 0.012 |  |
| 65p15 | PREDICTED: similar to glycogen synthase kinase 3 beta (LOC557882) | U | 0.211 | | -0.680 |  |
| 14k04 | malate dehydrogenase 1a NAD (soluble) (mdh1a) | D | -0.579 | | 0.205 |  |
| 14m01 | malate dehydrogenase 1b NAD (soluble) (mdh1b) | D | -0.822 | | 0.484 |  |
| 25j21 | 3-ketodihydrosphingosine reductase (kdsr) | U | 0.371 | | -0.424 |  |
| 28p08 | lysocardiolipin acyltransferase (lycat) | U | 0.353 | | -0.215 |  |
| 64n22 | PREDICTED: similar to Phospholipase D1 (PLD 1) (Choline phosphatase 1) (Phosphatidylcholine-hydrolyzing phospholipase D1) (hPLD1) (LOC799278) | U | 0.458 | | -0.442 |  |
| 80m06 | PREDICTED: similar to Phospholipase DDHD2 (DDHD domain-containing protein 2) (SAM WWE and DDHD domain-containing protein 1) (LOC565769) | D | -0.103 | | 0.312 |  |
| 59f19 | ATP synthase H+ transporting mitochondrial F0 complex subunit c (subunit 9) (atp5g) nuclear gene encoding mitochondrial protein | D | -0.383 | | 0.848 |  |
| 82j09 | ATP synthase H+ transporting mitochondrial F1 complex alpha subunit 1 cardiac muscle (atp5a1) nuclear gene encoding mitochondrial protein | D | -0.540 | | 0.575 |  |
| 59l20 | ATP synthase H+ transporting mitochondrial F1 complex gamma polypeptide 1 (atp5c1) nuclear gene encoding mitochondrial protein | D | -0.297 | | 0.417 |  |
| 39d09 | ATP synthase H+ transporting mitochondrial F1 complex O subunit (atp5o) nuclear gene encoding mitochondrial protein | D | -0.094 | | 0.759 |  |
| 62g09 | COX16 cytochrome c oxidase assembly homolog (S. cerevisiae) (cox16) | U | 0.532 | | -0.438 |  |
| 52n22 | isocitrate dehydrogenase 2 (NADP+) mitochondrial (idh2) nuclear gene encoding mitochondrial protein | D | -0.389 | | 0.510 |  |
| 40e09 | PREDICTED: similar to ATP synthase H+ transporting mitochondrial F0 complex subunit c (subunit 9) (LOC798840) | 5 | -0.273 | | 1.398 |  |
|  |  |  |  | |  |  |
| **Muscle structure and function** | |  |  | |  |  |
| 66o07 | actin alpha 2 smooth muscle aorta (acta2) | U | 0.885 | | -0.286 |  |
| 30h14 | alpha-tropomyosin (tpma) | D | -0.698 | | 0.336 |  |
| 09g12 | ARP3 actin-related protein 3 homolog (yeast) (actr3) | U | 0.546 | | -0.219 |  |
| 25p03 | ATPase Ca++ transporting cardiac muscle slow twitch 2a (atp2a2a) | D | -0.246 | | 0.303 |  |
| 60n06 | ATPase Ca++ transporting plasma membrane 4 (atp2b4) | U | 0.282 | | -0.349 |  |
| 61f07 | ATPase Na+/K+ transporting beta 2a polypeptide (atp1b2a) | U | 0.486 | | -0.071 |  |
| 08g05 | ba1 globin like (ba1l) | U | -0.013 | | -0.497 |  |
| 71b10 | bactin2 (bactin2) | 12 | 0.793 | | 0.278 |  |
| 14d03 | capping protein (actin filament) muscle Z-line beta (capzb) | U | 0.636 | | 0.041 |  |
| 20g12 | cofilin 2 like (cfl2l) | D | -0.246 | | 0.366 |  |
| 82k10 | coronin actin binding protein 2A (coro2a) | D | -0.288 | | 0.544 |  |
| 79p22 | dynactin 1a (dctn1a) | U | 0.452 | | -0.162 |  |
| 40l11 | myoglobin (mb) | D | -0.474 | | 0.676 |  |
| 40g13 | myosin binding protein C cardiac (mybpc3) | D | -0.671 | | 0.141 |  |
| 30i16 | myosin light polypeptide 2 skeletal muscle (mylz2) | D | -0.361 | | 0.354 |  |
| 26a20 | myosin light polypeptide 9 like (myl9l) | D | -0.158 | | 0.631 |  |
| 33e13 | prothymosin alpha a (ptmaa) | D | -0.268 | | 0.247 |  |
| 30g09 | prothymosin alpha b (ptmab) | D | -0.447 | | 0.378 |  |
| 51g18 | ryanodine receptor 1b (skeletal) (ryr1b) | D | -0.266 | | 0.259 |  |
| 29p21 | sarcoglycan epsilon (sgce) | U | 0.470 | | -0.351 |  |
| 76c20 | slow myosin heavy chain 3 (smyhc3) | 13 | -1.249 | | -0.214 |  |
| 66b22 | spectrin alpha 2 (spna2) | U | 0.583 | | -0.241 |  |
| 26c16 | titin-like (ttnl) | D | -0.603 | | -0.168 |  |
| 14d09 | tropomyosin 4 (tpm4) transcript variant 1 | D | -0.785 | | 0.347 |  |
| 30f18 | troponin T2 cardiac (tnnt2) | D | -0.653 | | 0.285 |  |
| 66b15 | troponin T3a skeletal fast (tnnt3a) | 7 | 1.044 | | -0.460 |  |
| 22b14 | PREDICTED: arrestin beta 1 (arrb1) | U | 0.220 | | -0.178 |  |
| 19d03 | PREDICTED: ryanodine receptor 3 (ryr3) | 5 | -0.207 | | 1.075 |  |
| 40c10 | PREDICTED: S100 calcium binding protein A10a (s100a10a) | D | -0.165 | | 0.078 |  |
| 54e24 | PREDICTED: similar to myosin heavy chain (LOC100008374) partial | 11 | -1.323 | | 0.065 |  |
| 25d21 | PREDICTED: similar to myosin I beta (LOC566459) | T | 0.078 | | -0.122 |  |
| 83m11 | PREDICTED: similar to myosin VIIA (LOC556141) | T | -0.462 | | -0.346 |  |
| 14h04 | PREDICTED: similar to myosin heavy polypeptide 2 fast muscle specific (LOC571931) | 11 | -1.326 | | 0.068 |  |
|  |  |  |  | |  |  |
| **Protein turnover** | |  |  | |  |  |
| 72h07 | elongation factor 1-alpha (ef1a) | D | -0.238 | | 0.285 |  |
| 12a09 | elongation factor-2 kinase (eef2k) | D | -0.782 | | -0.282 |  |
| 76n01 | eukaryotic translation elongation factor 2 like (eef2l) | D | -0.069 | | 0.602 |  |
| 76n02 | eukaryotic translation initiation factor 4e 1a (eif4e1a) | U | 0.398 | | -0.394 |  |
| 72l14 | phenylalanyl-tRNA synthetase alpha subunit (farsa) | U | 0.356 | | -0.395 |  |
| 12b01 | proteasome (prosome macropain) subunit alpha type5 (psma5) | D | -0.283 | | 0.679 |  |
| 16j16 | proteasome (prosome macropain) subunit beta type 9a (psmb9a) | D | -0.617 | | 0.478 |  |
| 39l13 | ribosomal protein L10 (rpl10) | D | -0.431 | | 0.296 |  |
| 78i24 | ribosomal protein L3 (rpl3) | D | -0.294 | | 0.224 |  |
| 29l04 | ribosomal protein L4 (rpl4) | D | -0.325 | | 0.090 |  |
| 52l12 | ribosomal protein L5b (rpl5b) | D | -0.225 | | 0.161 |  |
| 78k06 | ribosomal protein L6 (rpl6) | D | -0.218 | | 0.345 |  |
| 09e07 | ribosomal protein L8 (rpl8) | D | -0.221 | | 0.112 |  |
| 51i17 | ribosomal protein L9 (rpl9) | D | -0.289 | | 0.195 |  |
| 75n09 | ribosomal protein S2 (rps2) | D | -0.139 | | 0.453 |  |
| 78f20 | ribosomal protein S20 (rps20) | D | -0.213 | | 0.388 |  |
| 10l18 | ribosomal protein S8 (rps8) | D | -0.281 | | 0.016 |  |
| 02c15 | ribosomal protein large P0 (rplp0) | T | 0.110 | | 0.544 |  |
| 26b12 | ubiquinol-cytochrome c reductase core protein I (uqcrc1) | D | -0.239 | | 0.553 |  |
| 40b10 | ubiquinol-cytochrome c reductase complex III subunit VII (uqcrq) nuclear gene encoding mitochondrial protein | U | 0.378 | | 0.022 |  |
| 64b22 | ubiquitin A-52 residue ribosomal protein fusion product 1 (uba52) | D | -0.356 | | 0.240 |  |
| 54c08 | ubiquitin-conjugating enzyme E2 variant 2 (ube2v2) | U | 0.604 | | -0.071 |  |
| 52j19 | PREDICTED: proprotein convertase subtilisin/kexin type 9 (pcsk9) | 7 | 1.286 | | -0.022 |  |
| 06e03 | PREDICTED: similar to a disintegrin and metalloprotease domain 12 (LOC795199) | U | 0.513 | | -0.263 |  |
| 80l15 | PREDICTED: similar to ADAM metallopeptidase with thrombospondin type 1 motif 16 preproprotein (LOC568792) | 15 | 1.575 | | 0.184 |  |
| 12e24 | PREDICTED: similar to serine protease inhibitor Kazal type 2 (acrosin-trypsin inhibitor) (LOC100149146) | U | 0.377 | | -0.400 |  |
| 70b21 | PREDICTED: similar to UBiQuitin family member (ubq-1) (LOC558956) | U | 0.457 | | -0.033 |  |
| 80p05 | PREDICTED: similar to ubiquitin specific peptidase 2 (LOC100002673) | D | -0.120 | | 0.303 |  |
| 52p01 | PREDICTED: ubiquitin protein ligase E3 component n-recognin 5 (ubr5) | U | 0.230 | | -0.096 |  |
|  |  |  |  | |  |  |
| **Cellular regulatio**n | |  |  | |  |  |
| 53e11 | calmodulin 1a (calm1a) | D | -0.198 | | 0.299 |  |
| 28p23 | calmodulin 1b (calm1b) | D | -0.218 | | 0.189 |  |
| 34e17 | calmodulin 2a (phosphorylase kinase delta) (calm2a) | D | 0.135 | | 0.665 |  |
| 80a12 | calmodulin 2b (phosphorylase kinase delta) (calm2b) | D | -0.295 | | 0.600 |  |
| 29d16 | calmodulin 3a (phosphorylase kinase delta) (calm3a) | D | -0.194 | | 0.763 |  |
| 52g24 | calsequestrin 2 (casq2) | 10 | -1.245 | | 0.441 |  |
| 54a06 | DnaJ (Hsp40) homolog subfamily C member 18 (dnajc18) | U | 0.405 | | -0.358 |  |
| 58f19 | GLE1 RNA export mediator-like (gle1l) | U | 0.414 | | -0.481 |  |
| 55j06 | glutamate receptor interacting protein 1 (grip1) | U | 0.411 | | -0.488 |  |
| 78d24 | guanine nucleotide binding protein (G protein) beta polypeptide 1 (gnb1) | U | 0.417 | | -0.195 |  |
| 81a09 | guanine nucleotide binding protein (G protein) beta polypeptide 1 like (gnb1l) | D | -0.100 | | 0.123 |  |
| 09j22 | guanine nucleotide binding protein-like 3 (nucleolar) (gnl3) | D | -0.110 | | 0.519 |  |
| 67l06 | Janus kinase 1 (jak1) | U | 0.616 | | -0.291 |  |
| 54g09 | jun D proto-oncogene (jund) | U | 0.463 | | -0.291 |  |
| 39h06 | macrophage migration inhibitory factor (mif) | T | 0.355 | | -0.116 |  |
| 79o23 | MAP kinase-interacting serine/threonine kinase 2b (mknk2b) | 15 | 1.395 | | 0.086 |  |
| 22c12 | mitogen-activated protein kinase 12 (mapk12) | U | 0.795 | | -0.254 |  |
| 79b06 | mitogen-activated protein kinase 6 (mapk6) | U | 0.697 | | -0.163 |  |
| 22e10 | mitogen-activated protein kinase-activated protein kinase 3 (mapkapk3) | U | 0.391 | | -0.144 |  |
| 12o15 | N-myc downstream regulated gene 1 (ndrg1) transcript variant 1 | U | 0.913 | | -0.274 |  |
| 58b03 | N-myc downstream regulated gene 4 (ndrg4) | D | -0.537 | | -0.216 |  |
| 59p01 | phosphatase and tensin homolog A (ptena) | U | 0.421 | | -0.160 |  |
| 74j18 | PRKC apoptosis WT1 regulator like (pawrl) | 5 | -0.156 | | 0.818 |  |
| 66f17 | protein kinase C binding protein 1 like (prkcbp1l) | U | 0.705 | | -0.108 |  |
| 59d04 | protein kinase cAMP-dependent regulatory type II alpha B (prkar2ab) | U | 0.285 | | -0.341 |  |
| 30d09 | protein phosphatase 1 catalytic subunit beta isoform (ppp1cb) | U | 0.455 | | -0.137 |  |
| 78g11 | protein phosphatase 1 regulatory (inhibitor) subunit 14B (ppp1r14b) | U | 0.379 | | -0.255 |  |
| 40b17 | Rho GTPase activating protein 12 (arhgap12) | D | -0.207 | | 0.054 |  |
| 26g04 | signal transducing adaptor family member 2a (stap2a) | U | 0.272 | | -0.264 |  |
| 70b20 | T-cell activation GTPase activating protein (tagap) | U | 0.488 | | -0.322 |  |
| 79d11 | X-linked inhibitor of apoptosis (xiap) | U | 0.489 | | -0.047 |  |
| 62c04 | PREDICTED: Rho guanine nucleotide exchange factor (GEF) 10 (arhgef10) | U | 0.296 | | -0.522 |  |
| 07m05 | PREDICTED: similar to cell death activator CIDE-A (LOC558783) | T | -0.389 | | 0.235 |  |
| 54o04 | PREDICTED: similar to dual specificity phosphatase 8 (LOC100004581) | U | 0.228 | | -0.136 |  |
| 58i06 | PREDICTED: similar to EGF-like-domain multiple 3 (LOC557764) | U | 0.647 | | -0.664 |  |
| 62h14 | PREDICTED: similar to EGL nine (C.elegans) homolog 2 (LOC559569) | U | 0.358 | | -0.440 |  |
| 59p02 | PREDICTED: similar to egl nine homolog 1 (LOC100000346) | U | 0.794 | | -0.121 |  |
| 66k22 | PREDICTED: similar to GPI inositol-deacylase (Post-GPI attachment to proteins factor 1) (LOC100147850) | U | 0.505 | | -0.604 |  |
| 34b14 | PREDICTED: similar to GTPase IMAP family member 8 (LOC569567) | 12 | 0.787 | | 0.159 |  |
| 26g06 | PREDICTED: similar to Guanine nucleotide-binding protein G(q) subunit alpha (Guanine nucleotide-binding protein alpha-q) (LOC797978) | U | 0.286 | | -0.426 |  |
| 52h03 | PREDICTED: similar to mitogen-activated protein kinase kinase kinase 1 (predicted) (LOC564899) | U | 0.419 | | -0.245 |  |
| 10p06 | PREDICTED: similar to phosphatidic acid phosphatase type 2A (LOC794598) | T | -0.485 | | 0.005 |  |
| 62d20 | PREDICTED: similar to phosphodiesterase 11A (LOC561338) | U | 0.181 | | -0.503 |  |
| 58d06 | PREDICTED: similar to protein kinase lysine deficient 1 (LOC555647) | U | 0.441 | | -0.114 |  |
| 52p02 | PREDICTED: similar to protein tyrosine phosphatase gamma (LOC569183) | U | 0.756 | | -0.233 |  |
| 60p19 | PREDICTED: similar to protein tyrosine phosphatase receptor type sigma transcript variant 1 (LOC570717) | U | 0.792 | | -0.324 |  |
| 81i14 | PREDICTED: similar to protein tyrosine phosphatases epsilon (LOC567443) | U | 0.536 | | -0.254 |  |
| 15e10 | PREDICTED: similar to protein-kinase interferon-inducible double stranded RNA dependent inhibitor repressor of (P58 repressor) transcript variant 2 (LOC100151084) | 12 | 1.032 | | -0.075 |  |
| 54n16 | PREDICTED: similar to Rho GTPase activating protein 20 (LOC562063) | U | 0.892 | | -0.128 |  |
| 62k14 | PREDICTED: similar to Rho GTPase activating protein 21 (LOC556095) | U | 0.720 | | -0.756 |  |
| 72e19 | PREDICTED: similar to Rho GTPase activating protein 24 (LOC100005116) | 14 | -0.197 | | -0.459 |  |
| 79p16 | PREDICTED: similar to serine/threonine kinase receptor type1 (LOC559222) | U | 0.694 | | -0.094 |  |
| 21b06 | PREDICTED: similar to Serine/threonine-protein kinase Pim-3 (LOC560963) | D | -0.396 | | -0.099 |  |
| 63m01 | PREDICTED: similar to src-family tyrosine kinase SCK (LOC569951) | U | 0.497 | | -0.440 |  |
| 80a11 | PREDICTED: similar to very large inducible GTPase 1 (LOC558464) | U | 0.110 | | -0.383 |  |
| 81p08 | eph-like kinase 3 (ek3) | U | 0.483 | | -0.185 |  |
| 81b05 | F-box protein 9 (fbxo9) transcript variant 2 | U | 0.558 | | -0.200 |  |
| 58b08 | glucosamine (UDP-N-acetyl)-2-epimerase/N-acetylmannosamine kinase (gne) | U | 0.804 | | -0.266 |  |
| 58g04 | platelet-derived growth factor receptor-like (pdgfrl) | U | 0.592 | | -0.178 |  |
| 54l14 | PREDICTED: similar to tubby like protein 1 (LOC571108) | U | 0.484 | | -0.190 |  |
|  |  |  |  | |  |  |
| **Nuclear regulation** | |  |  | |  |  |
| 59d23 | activating transcription factor 1 (atf1) | U | 0.548 | | -0.176 |  |
| 30h22 | activating transcription factor 4 (tax-responsive enhancer element B67) (atf4) | 12 | 0.857 | | -0.030 |  |
| 12p10 | CCAAT/enhancer binding protein (C/EBP) beta (cebpb) | 2 | 1.151 | | -0. 04 |  |
| 66h14 | CCR4-NOT transcription complex subunit 6-like (cnot6l) | U | 0.983 | | -0.329 |  |
| 62c16 | general transcription factor IIIAa (gtf3aa) | U | 0.616 | | 0.005 |  |
| 54o20 | heterogeneous nuclear ribonucleoprotein U (hnrnpu) transcript variant 2 | U | 0.160 | | -0.498 |  |
| 27p23 | high-mobility group box 1 (hmgb1) | 10 | -1.056 | | 0.269 |  |
| 61n08 | high-mobility group box 3b (hmgb3b) | D | -0.441 | | 0.346 |  |
| 55g18 | metal-regulatory transcription factor 1 (mtf1) | U | 0.578 | | -0.133 |  |
| 14b04 | nuclear autoantigenic sperm protein (histone-binding) (nasp) | D | -0.454 | | 0.148 |  |
| 22o15 | nuclear factor of kappa light polypeptide gene enhancer in B-cells inhibitor alpha a (nfkbiaa) | U | 0.863 | | -0.247 |  |
| 67o01 | nuclear factor erythroid-derived 2 (nfe2) | U | 0.874 | | -0.263 |  |
| 19e01 | nuclear receptor subfamily 1 group D member 2a (nr1d2a) | D | -0.475 | | 0.556 |  |
| 78j09 | polymerase (RNA) II (DNA directed) polypeptide E (polr2e) | U | 0.154 | | -0.478 |  |
| 83l22 | polymerase I and transcript release factor (ptrf) transcript variant 2 | D | -0.589 | | 0.516 |  |
| 59h08 | proline-rich nuclear receptor coactivator 2 (pnrc2) | T | 0.157 | | -0.010 |  |
| 52g16 | RD RNA binding protein (rdbp) | D | -0.775 | | 0.451 |  |
| 77a06 | regulation of nuclear pre- domain containing 2 (rprd2) | U | 0.359 | | -0.409 |  |
| 67d01 | ribonuclease P 14 subunit (rpp14) | U | 0.396 | | -0.357 |  |
| 28l19 | ring finger protein 128 (rnf128) | U | 0.443 | | -0.254 |  |
| 58a21 | ring finger protein 2 (rnf2) | U | 0.388 | | -0.160 |  |
| 76b08 | signal sequence receptor alpha (ssr1) | U | 0.510 | | -0.151 |  |
| 62c20 | signal transduction and activation of transcription 1a (stat1a) | 12 | 0.962 | | 0.162 |  |
| 19p07 | similar to Ras association (RalGDS/AF-6) and pleckstrin homology domains 1 (LOC560319) | U | 0.630 | | -0.259 |  |
| 39e10 | small nuclear ribonucleoprotein 40 (U5) (snrnp40) | U | 0.728 | | -0.131 |  |
| 58h22 | topoisomerase (DNA) I like (top1l) | T | 0.563 | | -0.314 |  |
| 52i02 | transcription factor AP-2 alpha (tfap2a) | D | -0.667 | | 0.408 |  |
| 62d08 | transcription factor AP-2 gamma (activating enhancer binding protein 2 gamma) (tfap2c) | U | 0.453 | | -0.346 |  |
| 34d09 | upf1 regulator of nonsense transcripts homolog (yeast) (upf1) | D | -0.342 | | 0.006 |  |
| 32p15 | Y box binding protein 1 (ybx1) transcript variant 1 | D | -0.504 | | 0.363 |  |
| 16k22 | Y box binding protein 1 (ybx1) transcript variant 2 | D | -0.234 | | 0.383 |  |
| 80l19 | zinc finger BED domain containing 4 (zbed4) | U | 0.446 | | -0.071 |  |
| 17f08 | PREDICTED: histone deacetylase 6 (hdac6) | 11 | -0.966 | | -0.027 |  |
| 34p09 | PREDICTED: novel protein similar to vertebrate nuclear factor I/C (CCAAT-binding transcription factor) (NFIC) (LOC100005568) | U | 0.406 | | -0.217 |  |
| 52h08 | PREDICTED: nuclear factor of kappa light polypeptide gene enhancer in B-cells inhibitor zeta (nfkbiz) | U | 0.475 | | -0.213 |  |
| 60i22 | PREDICTED: similar to C2-HC type zinc finger protein X-MyT1 (LOC796059) partial | D | -0.387 | | 0.519 |  |
| 28c14 | PREDICTED: similar to cysteine-serine-rich nuclear protein 3 (LOC560270) | 12 | 0.813 | | 0.017 |  |
| 66e04 | PREDICTED: similar to histone cluster 2 H2ab (LOC100001004) | U | 0.531 | | -0.280 |  |
| 21b01 | PREDICTED: similar to muscle specific ring finger protein 1 (LOC100149610) | D | -0.110 | | -0.023 |  |
| 76p11 | PREDICTED: similar to ring finger protein 31 (LOC567642) | 9 | -0.387 | | 0.451 |  |
| 66c01 | PREDICTED: similar to TOX high mobility group box family member 3 (LOC555286) | U | 0.551 | | -0.147 |  |
| 14j04 | PREDICTED: similar to Transcription cofactor vestigial-like protein 1 (Vgl-1) (Protein TONDU) (LOC558513) | D | -0.802 | | 0.218 |  |
| 81j08 | PREDICTED: similar to zinc finger CCCH type containing 12A (LOC563108) | T | 0.420 | | -0.353 |  |
| 66p19 | PREDICTED: similar to zinc finger protein 180 (LOC100149538) | U | 0.608 | | -0.420 |  |
| 17c19 | PREDICTED: similar to zinc finger protein 3 (LOC100148086) | U | 0.626 | | -0.259 |  |
| 62b02 | PREDICTED: similar to zinc finger protein 569 (LOC100151570) | U | 0.497 | | -0.413 |  |
| 20a16 | PREDICTED: similar to Zinc finger protein 658 (LOC793732) | 11 | -1.117 | | -0.188 |  |
| 74h07 | PREDICTED: zinc finger ZZ-type with EF hand domain 1 (zzef1) | U | 0.461 | | -0.314 |  |
| 62j13 | FEZ family zinc finger 1 (fezf1) | U | 0.437 | | -0.005 |  |
| 67b20 | PREDICTED: similar to Probable RNA-directed DNA polymerase from transposon BS (Reverse transcriptase) (LOC100150846) | U | 0.304 | | -0.461 |  |
| 63m15 | PREDICTED: similar to Ribonuclease inhibitor (Ribonuclease/angiogenin inhibitor 1) (RAI) (Placental ribonuclease inhibitor) (RNase inhibitor) (RI) (LOC100149067) | U | 0.694 | | -0.259 |  |
|  |  |  |  | |  |  |
| **System-level regulation** | |  |  | |  |  |
| 08k24 | aryl hydrocarbon receptor 2 (ahr2) | T | -0.395 | | 0.062 |  |
| 62n04 | aryl-hydrocarbon receptor repressor a (ahrra) | U | 0.695 | | -0.344 |  |
| 35b10 | deiodinase iodothyronine type I (dio1) | D | -0.181 | | 0.264 |  |
| 58e05 | estrogen-related receptor alpha (esrra) | U | 0.470 | | 0.191 |  |
| 66j12 | fibroblast growth factor 1 (fgf1) | U | 0.530 | | -0.274 |  |
| 52j15 | fibroblast growth factor 13 (fgf13) | U | 0.337 | | -0.372 |  |
| 09b24 | glycoprotein hormones alpha polypeptide (cga) | U | 0.611 | | -0.096 |  |
| 66f10 | inhibitor of growth family member 1 (ing1) | U | 0.517 | | -0.373 |  |
| 78f08 | oxysterol binding protein-like 1A (osbpl1a) | U | 0.608 | | -0.468 |  |
| 66p04 | similar to adenosine A1 receptor (LOC100150997) | U | 0.739 | | -0.382 |  |
| 53e21 | similar to Metabotropic glutamate receptor 3 precursor (mGluR3) (LOC565256) | U | 0.388 | | 0.243 |  |
| 23b22 | TNF receptor-associated factor 1 (traf1) | U | 0.619 | | -0.234 |  |
| 26b22 | transducer of ERBB2 1a (tob1a) | D | -0.198 | | -0.175 |  |
| 61b24 | PREDICTED: GABA receptor alpha 6 subunit (gabara6) | T | 0.165 | | -0.008 |  |
| 13a11 | PREDICTED: similar to gamma-aminobutyric acid receptor associated protein (LOC793200) | T | -0.107 | | 0.073 |  |
| 34d06 | PREDICTED: similar to insulin receptor substrate 2 (LOC572001) | U | 0.755 | | -0.178 |  |
| 62d01 | inhibin beta Aa (inhbaa) | U | 0.321 | | -0.229 |  |
|  |  |  |  | |  |  |
| **Stress responses** | |  |  | |  |  |
| 25e05 | 2-aminoethanethiol (cysteamine) dioxygenase a (adoa) | U | 0.578 | | -0.161 |  |
| 63m14 | C1GALT1-specific chaperone 1 (c1galt1c1) | U | 0.860 | | -0.438 |  |
| 32o18 | heat shock protein alpha-crystallin-related 1 (hspb1) | U | 0.647 | | 0.126 |  |
| 28g08 | peroxiredoxin 5 (prdx5) nuclear gene encoding mitochondrial protein | U | 0.514 | | -0.187 |  |
| 59k23 | chaperonin containing TCP1 subunit 7 (eta) (cct7) | D | -0.451 | | 0.410 |  |
| 79p10 | cold inducible RNA binding protein (cirbp) transcript variant 1 | D | -0.094 | | -0.005 |  |
| 78g20 | cold inducible RNA binding protein (cirbp) transcript variant 2 | D | -0.283 | | 0.352 |  |
| 18o22 | heat shock protein family alpha-crystallin-related b7 (hspb7) | D | -0.356 | | 0.642 |  |
| 05c01 | stress-induced-phosphoprotein 1 (Hsp70/Hsp90-organizing protein) (stip1) | 4 | -1.104 | | 0.821 |  |
| 14k03 | heat shock protein 90kDa alpha (cytosolic) class B member 1 (hsp90ab1) | T | -0.300 | | 0.147 |  |
| 23f12 | heat shock protein alpha-crystallin-related b2 (hspb2) | T | -0.268 | | 0.017 |  |
| 55e23 | hypoxia up-regulated 1 (hyou1) | T | 0.181 | | -0.583 |  |
|  |  |  |  | |  |  |
| **Transport** |  |  |  | |  |  |
| 80m05 | chloride intracellular channel a (clica) | U | 0.768 | | 0.145 |  |
| 77g18 | novel protein similar to vertebrate solute carrier family 7 (cationic amino acid transporter y+ system) (LOC566469) | U | 0.731 | | -0.245 |  |
| 70n02 | solute carrier family 11 (proton-coupled divalent metal ion transporters) member 2 (slc11a2) | U | 0.293 | | -0.507 |  |
| 58n21 | solute carrier family 25 (carnitine/acylcarnitine translocase) member 20 (slc25a20) | U | 0.738 | | -0.274 |  |
| 62o10 | solute carrier family 25 (mitochondrial carrier; phosphate carrier) member 3 (slc25a3) | U | 0.513 | | -0.579 |  |
| 80a16 | solute carrier family 25 (mitochondrial carrier; phosphate carrier) member 3 like (slc25a3l) | U | 0.361 | | -0.041 |  |
| 66h20 | PREDICTED: similar to calcium channel voltage-dependent beta 4b subunit (LOC564197) partial | U | 0.830 | | -0.546 |  |
| 09d06 | PREDICTED: similar to proton/amino acid transporter 4 (LOC564174) | U | 0.526 | | -0.108 |  |
| 66m03 | PREDICTED: similar to solute carrier family 25 member 29 (LOC100006658) | U | 0.517 | | -0.349 |  |
| 27o20 | PREDICTED: solute carrier family 6 (neurotransmitter transporter creatine) member 8 (slc6a8) | U | 0.661 | | -0.419 |  |
| 32n13 | solute carrier family 25 (mitochondrial carrier Aralar) member 12 (slc25a12) | D | -0.559 | | 0.243 |  |
| 26i08 | solute carrier family 25 (mitochondrial carrier; adenine nucleotide translocator) member 4 (slc25a4) | D | -0.845 | | 0.712 |  |
| 80p08 | solute carrier family 25 alpha member 5 (slc25a5) | D | -0.487 | | 0.637 |  |
| 28j06 | voltage-dependent anion channel 2 (vdac2) | D | -0.622 | | 0.737 |  |
| 25f20 | PREDICTED: solute carrier family 7 (cationic amino acid transporter y+ system) member 9 (slc7a9) | D | -0.211 | | 0.673 |  |
| 14f04 | PREDICTED: solute carrier family 2 (facilitated glucose/fructose transporter) member 5 (slc2a5) | 13 | -0.982 | | -0.327 |  |
| 08j22 | PREDICTED: similar to solute carrier family 46 member 3 (LOC556497) | T | -0.388 | | 0.463 |  |
