# Supplementary material for: Life without Oxygen: Gene Regulatory Responses of the Crucian Carp (Carassius carassius) Heart Subjected to Chronic Anoxia
Source: PLoS One. 2014 Nov 5;9(11):e109978. doi: 10.1371/journal.pone.0109978 (PMC4220927; doi:10.1371/journal.pone.0109978)
Supplement: Table S2 — List of 291 genes selected from the 777 judged as being differentially expressed in at least one of the indicated contrasts in the experimental design in Fig 1 , together with those responding to temperature. The genes have been collated into broad functional groups. The ‘cluster’ column indicates to which cluster in Fig 4 of the accompanying paper each gene belonged – U indicates anoxia up-regulated genes, D- anoxia downregulated genes in Figure 4B while cluster 1–16 indicates to which outlier group in Fig 4D. T- indicates temperature-regulated genes not shown in Fig 4. We show the log2 ratio for two contrasts, as indicated. Not listed are genes lacking a meaningful functional annotation, and others from other functional categories. (DOCX) [file pone.0109978.s006.docx]

Supplementary Table S1

| Clone i.d. | Gene name, *p* and *q* | 13A1/13N7 | | 13A7/13N7 | | | 13A7R7/13N7 |
| --- | --- | --- | --- | --- | --- | --- | --- |
|  | Hydroxyacylglutathione hydrolase cytoplasmic |  |  | | |  | |
| 01a03 | Log_2_fold change | 0.812 | 0.955 | | | 0.324 | |
|  | *p* value (Student’s t test) | 0.051 | 0.023 | | 0.419 | | |
|  | *q* value (FDR) | 0.577 | 0.295 | | 0.748 | | |
|  |  |  |  | |  | | |
